# Comparative genomic analysis of multiple strains of *two* unusual plant pathogens: *Pseudomonas corrugata* and *Pseudomonas mediterranea*

**DOI:** 10.3389/fmicb.2015.00811

**Published:** 2015-08-07

**Authors:** Emmanouil A. Trantas, Grazia Licciardello, Nalvo F. Almeida, Kamil Witek, Cinzia P. Strano, Zane Duxbury, Filippos Ververidis, Dimitrios E. Goumas, Jonathan D. G. Jones, David S. Guttman, Vittoria Catara, Panagiotis F. Sarris

**Affiliations:** ^1^Plant Biochemistry and Biotechnology Laboratory, Department of Agriculture, School of Agriculture and Food Technology, Technological Educational Institute of CreteHeraklion, Greece; ^2^Science and Technology Park of SicilyCatania, Italy; ^3^School of Computing, Federal University of Mato Grosso do SulCampo Grande, Brazil; ^4^The Sainsbury Laboratory, John Innes CentreNorwich, UK; ^5^Department of Agriculture, Food and Environment, University of CataniaCatania, Italy; ^6^Plant Pathology and Bacteriology Laboratory, Department of Agriculture, School of Agriculture and Food Technology, Technological Educational Institute of CreteHeraklion, Greece; ^7^Centre for the Analysis of Genome Evolution & Function, University of TorontoToronto, ON, Canada

**Keywords:** comparative genomics, type III secretion system, type VI secretion system, effectors, siderophores, polyketides, non-ribosomal peptides, pith necrosis

## Abstract

The non-fluorescent pseudomonads, *Pseudomonas corrugata* (*Pcor*) and *P. mediterranea* (*Pmed*), are closely related species that cause pith necrosis, a disease of tomato that causes severe crop losses. However, they also show strong antagonistic effects against economically important pathogens, demonstrating their potential for utilization as biological control agents. In addition, their metabolic versatility makes them attractive for the production of commercial biomolecules and bioremediation. An extensive comparative genomics study is required to dissect the mechanisms that *Pcor* and *Pmed* employ to cause disease, prevent disease caused by other pathogens, and to mine their genomes for genes that encode proteins involved in commercially important chemical pathways. Here, we present the draft genomes of nine *Pcor* and *Pmed* strains from different geographical locations. This analysis covered significant genetic heterogeneity and allowed in-depth genomic comparison. All examined strains were able to trigger symptoms in tomato plants but not all induced a hypersensitive-like response in *Nicotiana benthamiana*. Genome-mining revealed the absence of type III secretion system and known type III effector-encoding genes from all examined *Pcor* and *Pmed* strains. The lack of a type III secretion system appears to be unique among the plant pathogenic pseudomonads. Several gene clusters coding for type VI secretion system were detected in all genomes. Genome-mining also revealed the presence of gene clusters for biosynthesis of siderophores, polyketides, non-ribosomal peptides, and hydrogen cyanide. A highly conserved quorum sensing system was detected in all strains, although species specific differences were observed. Our study provides the basis for in-depth investigations regarding the molecular mechanisms underlying virulence strategies in the battle between plants and microbes.

## Background

*Pseudomonas corrugata* (*Pcor*) Robert and Scarlet 1981 emend. Sutra et al. (1997) and *P. mediterranea* (*Pmed*) Catara et al. (2002) are two closely related Gammaproteobacteria species belonging to the genus *Pseudomonas sensu stricto* (De Vos et al., 1985; Kersters et al., 1996). This genus predominantly consists of fluorescent species, but also contains some non-fluorescent species, including, *P. stutzeri, P. alcaligenes, P. pseudoalcaligenes, P. mendocina, P. lemoignei, P. ruhlandii, P. frederiksbergensis, P. graminis, P. plecoglossicida, Pcor*, and *Pmed*.

*Pcor* was the first of the two species discovered as the causal agent of tomato pith necrosis (TPN) (Scarlett et al., 1978; Catara, 2007). It has since been isolated from several other crop plants suffering pith necrosis, including pepper (Lopez et al., 1988), chrysanthemum (Fiori, 1992) and geranium (Magyarosy and Buchanan, 1995). *Pcor* is ubiquitous and has been isolated from the root environment in different countries (Kovacevich and Ryder, 1991; Ryder and Rovira, 1993; Schisler and Slininger, 1994; Achouak et al., 2000; Walker et al., 2000; Pandey et al., 2001).

Phylogenetic analyses based on 16S rRNA gene sequences placed *Pcor* within the *P. fluorescens* branch (Moore et al., 1996; Anzai et al., 2000), whereas, in another study of the combined nucleotide sequences of the *rpoD* and *gyrB* genes, the bacterium was included in intrageneric cluster II within the “*P. fluorescens* complex” (*P. syringae* and *P. putida* being the other two) (Yamamoto et al., 2000). A polyphasic approach revealed that two closely related but distinct taxa were present within the *Pcor* species. The taxon which contained the type strain CFBP2431T maintained the epithet *Pcor*, while the other strains included were assigned to a novel species, named *P. mediterranea* (Catara et al., 2002). The two species are phenotypically distinguishable by the ability of *Pcor*, but not of *Pmed*, to utilize histamine, 2-ketogluconate and meso-tartrate as a unique carbon source. The two species can also be clearly distinguished by 16S rDNA analysis, by means of DNA-based fingerprinting methods (Catara et al., 2000, 2002) and by multi-locus sequence analysis (MLSA) (Trantas et al., 2015). Multiplex, conventional, or real-time PCR can also be used to screen tomato planting materials for detection of, and discrimination between, the two bacterial species (Catara et al., 2000; Licciardello et al., 2011). Moreover, a phylogenetic analysis of the concatenated sequences of four core “housekeeping” genes (16S rRNA, *gyrB, rpoB*, and *rpoD*) of 107 type strains of *Pseudomonas* species placed *Pcor* and *Pmed* in a separate subgroup within the *P. fluorescens* lineage (Mulet et al., 2010).

Pith necrosis is characterized by the necrosis and hollowing of the parenchymatic tissue of the stem. Usually, the first visible symptom is chlorosis and withering of the youngest leaves, followed by loss of turgor and eventual collapse of the whole plant in later stages of the disease. The disease occurs world-wide in all tomato-growing areas and can cause severe crop losses. *Pcor* is a demonstrated plant pathogen, but has potential to be used as a biocontrol agent (Catara, 2007). Most strains have been used as biocontrol agents against different plant pathogenic fungi. Many strains, including those responsible for pith necrosis, have demonstrated either *in vitro* or *in vivo* antimicrobial activity.

*Pcor* produces the antimicrobial and phytotoxic cyclic lipopeptides (CLPs) cormycin A, corpeptin A, and corpeptin B (Emanuele et al., 1998; Scaloni et al., 2004). Corpeptins were isolated from the culture filtrates of the *Pcor* type strain NCPPB2445 (Emanuele et al., 1998). The corpeptin peptide (CP) moiety showed similarity to *Pseudomonas* peptins such as syringopeptins (SP22s, SP25s), fuscopeptins (FPs), and tolaasins (ToI-A). Since a number of phytopathogenic *Pseudomonas* spp. produce antimicrobial nonapeptides, Scaloni screened the culture filtrates of a number of *Pcor* strains and demonstrated that nonapeptide production is strain-dependent (Scaloni et al., 2004). In the same study Cormycin A was also characterized as a novel compound from the culture filtrates of the strain IPVCT 10.3. Cormycin and corpeptins play a pivotal role in *Pcor* and *Pmed* virulence. Mutants unable to produce one or both of them are still able to colonize tomato stem parenchymatic tissues but the symptoms associated with pith necrosis are significantly reduced (Licciardello et al., 2012; Strano et al., 2014).

Recent investigations on the interactions of both *Pcor* and *Pmed* with plants highlight the pivotal role of quorum sensing in the regulation of virulence traits (Licciardello et al., 2007, 2012, 2009). Both *Pcor* and *Pmed* have a quorum sensing system mediated by N-acyl homoserine lactone (AHL-QS) signal molecules. Strains of both species from different geographical locations and plant origins produce the same AHLs at comparable levels (Licciardello et al., 2007, 2012). In plant-associated *Pseudomonas* the AHL-QS has been shown to have a role in virulence of plant pathogenic species and in the regulation of traits involved in biological control activity (Quiñones et al., 2004, 2005; Venturi, 2006; Wei and Zhang, 2006; Licciardello et al., 2007, 2012; Hosni et al., 2011; Mattiuzzo et al., 2011). Specifically in *Pcor* and *Pmed*, AHL-QS and the genetically linked transcriptional regulator RfiA have a role in virulence in tomato (Licciardello et al., 2009, 2012) and in corpeptin production (Strano et al., 2014).

Here, we present the draft genome sequences of five *Pmed* and four *Pcor* strains, including the type strains, in order to investigate mechanisms of pathogenicity and the synthesis of antimicrobial compounds. The selected strains were isolated from different geographical locations in order to cover the genetic heterogeneity of the two species, allowing us to perform in-depth comparative genomic analysis. Our analysis revealed unique features of the pathogenicity of *Pcor* and *Pmed*, and significant insights into the antimicrobial activity and production of secondary metabolites in these species.

## Materials and methods

### Bacterial strains and genomic DNA preparation

We compared the genomes of nine strains: four *Pcor* strains and five *Pmed* strains. Table 1 contains the metadata of all strains. Strains were selected based on their geographical origin (Greece, Italy, Spain, and U.K.) and intraspecific diversity based on phenotypic and genotypic characteristics reported in previous studies (Catara et al., 2002; Trantas et al., 2015). The type strains of both species were included. The genome set included seven *de novo* sequenced strains and two previously sequenced strains: *Pcor* CFBP5454 (Licciardello et al., 2014b) and *Pmed* CFBP5447 (Licciardello et al., 2014a). The strains were grown at 28°C either in Nutrient Dextrose agar (NDA) or Lysogeny Broth (LB) agar.

**Table 1:** **Strains used for the comparative analysis of *Pseudomonas corrugata* (*Pcor*) and *P. mediterranea* (*Pmed*)**.

**Strain**	**Species**	**Isolation source**	**Location of isolation**	**IMG genome ID**	**References**
TEIC1022	*Pmed*	Infected tomato plant	Crete, Greece	2563366521	Trantas et al., 2015
TEIC1105	*Pmed*	Infected tomato plant	Crete, Greece	2563366524	Trantas et al., 2015
CFBP5404	*Pmed*	Infected pepper plant	Tenerife, Spain	2563366522	Catara et al., 2002
CFBP5444	*Pmed*	Infected tomato plant	Sicily, Italy	2563366526	Catara et al., 2002
CFBP5447T	*Pmed*	Infected tomato plant	Sicily, Italy	2596583589	Catara et al., 2002
TEIC1148	*Pcor*	Infected tomato plant	Crete, Greece	2563366520	Trantas et al., 2015
CFBP5403	*Pcor*	Infected tomato plant	Tenerife, Spain	2563366523	CFBP
CFBP5454	*Pcor*	Infected tomato plant	Sicily, Italy	2558309045	Catara et al., 2002
NCPPB2445T	*Pcor*	Infected tomato plant	UK	2563366525	NCPPB

*TEIC, Technological Educational Institute of Crete Bacterial collection; CFBP, International Center for Microbial Resources, French Collection for Plant-associated Bacteria, INRA, Angers, France; NCPPB, National Collection of Plant Pathogenic Bacteria, Fera, York, U.K*.

DNA was extracted from over-night cultures in LB broth using the DNeasy Blood & Tissue Kit from QIAGEN (UK) according to the manufacturer's instructions.

### Phenotypic characterization and *In Planta* tests

*Pcor* and *Pmed* strains were tested in assays that had been previously reported to reveal phenotypic differences between species (Catara et al., 1997, 2002; Sutra et al., 1997; Solaiman et al., 2005). To determine the production of protease, lipase, and gelatinase, strains were seeded on agar plates containing the appropriate substrates as previously described (Lelliott and Stead, 1987; Schaad et al., 1988).

Antimicrobial activity of *Pcor* and *Pmed* was assessed on potato dextrose agar (PDA), against two CLP indicator microorganisms (the Gram positive bacterium *Bacillus megaterium* ITM100 and the yeast *Rhodotorula pilimanae* ATTC26432) and against two phytopathogenic bacteria (*P. syringae* pv. tomato PVCT28.3.1 and *Xanthomonas campestris* pv. *campestris* PVCT 62.4) (Licciardello et al., 2007, 2012). The antimicrobial activity of culture filtrates of selected strains was assessed by the well-diffusion assay against the CLP indicator strains as described by Licciardello et al. (2012). Culture filtrates were prepared from 4 day-old still bacterial cultures in Improved Minimal Medium (IMM) (Surico et al., 1988). After centrifugation (9000 g, 20 min), the supernatant was passed through a 0.22 μm Millipore filter (Millipore, Billerica, MA, U.S.A.) to obtain cell-free culture filtrates that were 10 × concentrated using the Vacufuge concentrator 5301 (Eppendorf, Milan, Italy). All tests were carried out twice and with three technical replicates each time.

The hypersensitive response (HR) test was performed using a blunt syringe to infiltrate 10^8^ CFU ml^−1^ of bacteria into leaves of *Nicotiana benthamiana*. Ten leaf panels were infiltrated per strain. After infiltration, plants were placed at 25°C in a growth chamber and the collapse/necrosis of the mesophyll was assessed daily. *Nicotianna benthamiana* seeds kindly provided by Prof. N. Panopoulos (University of Crete, Greece).

Artificial inoculations were performed in tomato (commercial hybrid Elpida), and pepper (commercial hybrid Raiko) plants at the stage of three or four true leaves. Inoculations were made with 24–48 h bacterial cultures grown on KB medium. The inoculum was collected with a sterile toothpick and used for inoculation by piercing the stems of the plants with a lateral tilt to a depth of 0.5 cm. The inoculated sites were covered with parafilm and the plants remained at high humidity (90–100%) at ambient temperature (about 23°C) for 48 h. Two days later the plants were transferred to a greenhouse with 60–70% humidity and 15–26°C temperature. Plants were observed for 10–15 days after inoculation (Catara et al., 2002; Trantas et al., 2015).

### Library preparation, sequencing, annotation and assembly

Genomic DNA was isolated from the strains TEIC1022, TEIC1105, CFBP5404, CFBP5444, TEIC1148, CFBP5403, and NCPPB2445T using the Gentra Puregene DNA Purification Kit (Qiagen). Illumina DNA library preparation was performed using the NEBNext DNA Library Master Mix kit (New England Biolabs) following the manufacturer's protocol for user supplied barcodes. The quality of the prep prior to sequencing was confirmed on a Bioanalyzer 2100 with a DNA1000 chip (Agilent) and a Qubit Fluorometer using a Quant-iTTM dsDNA BR assay kit (Life Technologies). Genome sequencing was performed on an Illumina MiSeq using V2 chemistry producing 150 base, paired-end reads. Read numbers per strain are reported in Supplementary File 1.

The draft genome sequences of *Pcor* strain CFBP5454 and *Pmed* strain CFBP5447T were previously obtained by using Illumina GAIIx technology combining mate-pair and paired-end libraries (CFBP5447) or paired-end libraries (CFBP5454) (Licciardello et al., 2014a,b) and used for comparative analyses. For this study, the 157 and 47 contigs of CFBP5454 and CFBP5447T strains were linked and placed into 84 and 36 scaffolds or super-contigs, respectively. The orientation, order and distance between the contigs were estimated using the insert size between the paired-end and/or mate-pair reads. The analysis was performed using the SSPACE Premium scaffolder version 2.0 (Boetzer et al., 2011). Gaps within scaffolds were automatically closed with GapFiller (Boetzer and Pirovano, 2012).

### Genome assemblies, gene mining, and alignments

*De novo* sequence assemblies were performed by CLC Genomics Workbench 7 (http://www.clcbio.com). All genomes were annotated through JGI's auto-annotation pipeline (Markowitz et al., 2014). All BLAST queries (Altschul et al., 1997) were accomplished at JGI (Mavromatis et al., 2009).

### Phylogenetic analyses

Whole genome phylogenetic trese were built using Orthologsorter (Farias and Almeida, 2013). All protein-coding genes were first analyzed by OrthoMCL (Li et al., 2003). Among all families found, those with exactly one representative in each genome were taken, except for out-group genomes, which were allowed to have zero or one gene in each family. Next, Muscle (Edgar, 2004) and Gblocks (Castresana, 2000) were used with the default parameters to align each chosen family and remove non-informative columns. Then, all alignments were concatenated, resulting in a complete alignment, which was then processed by RAxML (Stamatakis, 2006) to build the tree, using the PROTCAT model, rapid bootstrapping and a subsequent maximum likelihood (ML) search.

Gene-specific phylogenetic analysis of the following genes was undertaken: *fruK* (fructose-1-phosphate kinase), *gltA* (type II citrate synthase), *gyrB* (b subunit of DNA gyrase), *mutL* (methyl-directed mismatch repair protein), *rpoB* (beta subunit of RNA polymerase), *rpoD* (sigma factor of RNA polymerase). Sequences were extracted from our assemblies of each strain. Sequences for reference strains were retrieved from GenBank. Sequence alignments were carried out using CLUSTALW (Thompson et al., 1994) with the default parameters. The phylogenetic trees were generated in MEGA6 (Tamura et al., 2013). Neighbor-Joining trees were generated according to the p-distance method (Saitou and Nei, 1987). In order to confirm the relationships found with the Neighbor-Joining dendrogram, comparison Maximum Likelihood trees were generated according to the Tamura-Nei model (Tamura and Nei, 1993). The percentage of replicate trees (1500 replicates) in which the associated strains clustered together was estimated (Felsenstein, 1985) and is shown next to the tree nodes.

## Results and discussion

### Variable phenotypic traits

**Figure 1:**
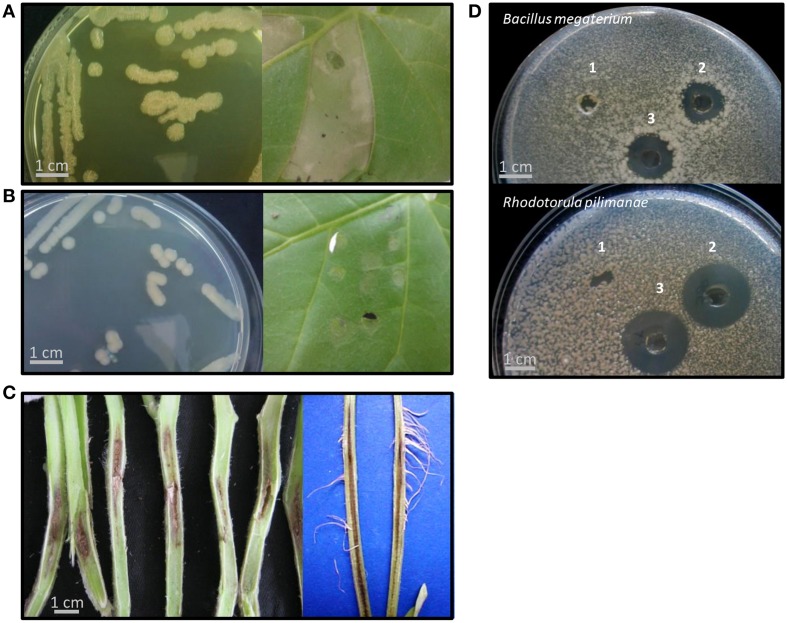
**Representative phenotypes of *Pseudomonas corrugata* (*Pcor*) and *P. mediterranea* (*Pmed*) strains in this study**. **(A,B)** Strains showed either a rough colony morphology with production of a yellow diffusible pigment in the medium (R-phenotype) or smooth colonies that did not produce pigment (S-phenotype). All “R” strains induced a hypersensitive-like response (HR) in *Nicotiana benthamiana* (*Pcor* CFBP5454 in **A**) while the “S” strains *Pcor* TEIC1148 and Pmed CFBP5444 (the latter in **B**) did not induce HR. **(C)** All strains were able to induce tomato pith necrosis. **(D)** Representative bacterial culture filtrates produced in cyclic lipopeptide (CLP)-inducing conditions were tested for antimicrobial activity against the Gram-positive bacterium *Bacillus megaterium a*nd the yeast *Rhodotorula pilimanae*. *Pmed* CFBP5444 culture filtrate representative of the “S” phenotype did not show antimicrobial activity. 1, *Pmed* CFBP5444; 2, *Pcor* CFBP5454; 3, *Pmed* CFBP5447. All tests were performed at least three times with identical results.

The *Pcor* species epithet “*corrugata*” refers to the typical morphology of bacterial colonies on NDA; slightly raised, with a wrinkled-rough surface and undulate margins that produce a yellow diffusible pigment (Scarlett et al., 1978). *Pmed* strains also show this morphology. Nevertheless, in both species non-pigmented strains with smooth colonies and intermediate phenotypes have been observed (Siverio et al., 1993; Sutra et al., 1997; Catara et al., 2002). The strains assessed in this study (Table 1) were representative of this colony variability. Five of the strains showed the “rough-phenotype” and produced yellow diffusible pigments (R-type). Three strains shared a “smooth phenotype” with whitish-cream, smooth-surfaced colonies that did not produce any pigment (S-type; Figures 1A,B). One strain showed an intermediate phenotype (I-type), growing in smooth, slightly elevated colonies with a low-level of diffusible pigment (Table 2).

**Table 2:** **Features linked with antimicrobial activity of genomes under study**.

**Strain**	**Species**	**Morphology**	**Hydrolytic activity**			**HR**	**HCN**	**Antimicrobial activity against**			
			**Casein**	**Gelatine**	**Tween 80**			***Rp***	***Bm***	***Pst***	***Xcc***
NCPPB2445T	*Pcor*	R	W	W	+	+	W	+	+	+	−
TEIC1148	*Pcor*	S	−	−	−	−	−	+	−	−	−
CFBP5403	*Pcor*	R	+	+	−	+	+	+	+	+	−
CFBP5454	*Pcor*	R	+	+	+	+	+	+	+	+	+
CFBP5447T	*Pmed*	R	+	+	W	+	+	+	+	+	−
TEIC1022	*Pmed*	S	−	−	−	+	−	+	−	−	−
TEIC1105	*Pmed*	I	W	W	−	+	W	+	+	+	−
CFBP5404	*Pmed*	R	W	W	−	+	+	+	+	+	−
CFBP5444	*Pmed*	S	−	−	−	−	−	+	−	−	−

*Species abbreviations are Pcor, Pseudomonas corrugata; Pmed, P. mediterranea; Rp; Rhodotorula pilimanae; Bm, Bacillus megaterium; Pst, P. syringae pv. tomato and Xcc, Xanthomonas campestris pv. campestris. TEIC: Technological Educational Institute of Crete Bacterial collection; CFBP: International Center for Microbial Resources, French Collection for Plant-associated Bacteria, INRA, Angers, France; NCPPB, National Collection of Plant Pathogenic Bacteria, Fera, York, U.K. Isolate names followed by the letter “T” are type strains; R, “rough-phenotype” producing yellow diffusible pigments (R-type); S, “smooth phenotype” with whitish-cream smooth-surfaced colonies that do not produce any pigment (S-type); I, “intermediate phenotype” producing rather smooth colonies with weak diffusible pigment (I-type); HR, hypersensitive response-like reaction; HCN, production of hydrogen cyanide; +, positive reaction; −, negative reaction or no antimicrobial activity; w, weak-positive reaction*.

The appearance of pleiotropic mutants after sub-culturing or re-isolation from soil occurred and was observed as a conversion from rough to smooth colony morphology. This phenomenon was also associated with either loss of exoenzyme activity (i.e., inability to hydrolyse casein, gelatine, lecithin, and Tween 80), or modification of antagonistic activity and hypersensitive response (HR) induction (Siverio et al., 1993; Gustine et al., 1995; Barnett et al., 1999). In particular, we observed that the three S-type strains lacked caseinase and gelatinase activity, whereas the R- and I-type strains maintained the ability to hydrolyze casein and gelatin. On the other hand, only three R- strains were able to hydrolyze Tween 80 (lipase test; Table 2). It is noteworthy that at least one of the strains in this study (CFBP5444) was isolated from plants as a natural S phenotype.

An important trait of *Pcor* is that it elicits a plant defense response in non-host plants; namely, the production of the phytoalexin medicarpin in white clover callus and an HR-like response in tobacco leaves (Gustine et al., 1990, 1995). Tobacco HR-like response is not induced by all the strains of the two species (Sutra et al., 1997; Catara et al., 2002). We tested the ability of suspensions of *Pcor* and *Pmed* to induce an HR-like response in *N. benthamiana* leaves. Within 24 h of infiltration, seven out of the nine strains caused the infiltrated leaf panels to collapse, and after a further 24 h these leaf panels turned necrotic. Leaf panels inoculated with the S-type strains *Pcor* TEIC1148 and *Pmed* CFBP5444 did not show any symptoms (Figures 1A,B). However, no genes encoding components of the T3SS was found in any of the *Pcor* and *Pmed* genomes (see results for *P. corrugata* and *P. mediterranea* lack of T3SS). In a study carried out on *Pcor* CFBP5454 (which gives an HR-like response) it was observed that the bacterial population titre in infiltrated *N. benthamiana* leaf panels decayed rapidly after inoculation. On the other hand when leaf panels were infiltrated with non-producing CLP mutants (which does not give an HR-like response) the population titre was maintained (Strano et al., 2014). This suggested a role for CLPs in eliciting plant defense responses (Strano et al., 2014).

### Artificial inoculations

Tomato and pepper seedlings were *Pcor*-inoculated at the developmental stage of three-to-four true leaves and within 15 days showed the typical symptoms of pith necrosis disease (Figure 1C). Similar symptoms were observed on plants infected with *Pmed* strains. In all infected plants, the pith necrosis extended 1.5–3 cm on both sides of the initial infection point. Secondary acquired aerial roots appeared on some artificially inoculated tomato plants. Collectively, our results indicated no significant difference of pathogenic severity between *Pcor* and *Pmed* strains on tomato plants.

### Genome-wide sequence data

The genomic sequences of nine *Pcor* and *Pmed* strains (Table 1) were obtained using Illumina MiSeq or GAIIx technology. This produced between 1,209,534 and 11,800,202 paired-end reads (Table 3 and Supplementary File 1). *De novo* genome assembly for the nine strains was performed independently for each strain using the CLC Genomics Workbench (CLCbio, Aarhus, Denmark). The assemblies contain 84–442 contigs with an N75 ranging from 16,551 to 120,020. The total number of nucleotides assembled in contigs ranged from 6,084,011 to 6,266,776 for the *Pcor* strains and from 6,231,327 to 6,461,985 for *Pmed* strains.

**Table 3:** **Short table of genomic statistics for *Pseudomonas corrugata* and *P. mediterranea* strains under study**.

	***P. corrugata***				***P. mediterranea***				
	**CFBP5403**	**CFBP5454**	**NCPPB2445**	**TEIC1148**	**CFBP5404**	**CFBP5444**	**CFBP5447**	**TEIC1022**	**TEIC1105**
Total number of reads	11,800,202	8,420,692	1,519,930	8,608,470	1,209,534	1,394,308	24,635,686	1,276,402	23,866,648
Average read length	35	70.6	145	35	145	145	51	145	44
Total bases	413,007,070	594,525,036	220,389,850	301,296,450	175,382,430	202,174,660	1,256,357,465	185,078,290	1,040,233,200
DNA, total number of bases in contigs	6,166,582	6,195,615	6,084,011	6,266,776	6,281,542	6,298,862	6,270,666	6,231,327	6,299,426
Ambiguous bases	39,282	2299	3749	31,312	3143	3499	18	11,988	1,651
Percentage of ambiguous bases	0.64	0.04	0.06	0.50	0.05	0.06	0.00	0.19	0.03
DNA coding number of bases	5,471,555	5,491,990	5,391,923	5,567,198	5,564,235	5,572,667	5,573,461	5,507,149	5,573,467
DNA G+C number of bases	3,706,298	3,747,457	3,686,460	3,763,425	3,846,554	3,853,935	3,862,577	3,813,571	385,5401
GC content (%)	60.1	60.5	60.6	60.1	61.2	61.2	60.7	61.2	61.2
Total number of contigs	442	84	104	432	150	89	47	101	91
N75	17,365	48,599	85,837	16,551	11,1354	118,717	447,675	83,242	120,020
N50	29,882	79,475	202,143	32,179	188,131	229,164	662,701	142,363	230,696
Genes total number	5912	5570	5463	5845	5652	5638	5533	5594	5643
Coding density (%)	87.9	88.5	88.4	88.4	88.4	88.3	88.4	88.1	88.3
Completeness (%)	97.02	99.69	99.68	99.14	99.69	99.68	99.69	99.45	99.69
Contamination (%)	0.80	0.42	0.45	0.47	0.48	0.79	0.48	0.43	0.79
Protein coding genes	5809	5443	5313	5737	5515	5484	5786	5450	5481
RNA genes	103	127	150	108	137	154	135	144	162
Protein coding genes with function prediction	4812	4621	4516	4753	4608	4598	4804	4611	4606
Protein coding genes without function prediction	997	822	797	984	907	886	982	839	875
Protein coding genes for enzymes	1307	1355	1341	1331	1339	1339	1312	1337	1339
w/o enzymes but with candidate KO based enzymes	147	19	26	68	17	11	130	24	13
Protein coding genes connected to transporter classification	832	792	777	810	790	791	809	785	791
Protein coding genes connected to KEGG pathways	1497	1564	1546	1536	1565	1570	1523	1538	1570
Protein coding genes connected to KEGG Orthology (KO)	2733	2850	2807	2808	2833	2828	2760	2804	2828
Protein coding genes connected to MetaCyc pathways	1272	1325	1311	1300	1313	1313	1287	1312	1313
Protein coding genes with COGs	3839	4012	3927	3923	3965	3979	3927	3949	3978
Chromosomal cassettes	717	532	508	697	556	562	613	560	559
Biosynthetic clusters	39	46	38	45	49	50	44	46	43
Genes in biosynthetic clusters	289	386	332	331	397	410	389	405	369
COG clusters	2033	2051	2046	2053	2037	2039	2026	2026	2039
KOG clusters	841	841	852	852	837	839	840	845	839
Pfam clusters	2601	2566	2551	2597	2,547	2,551	2,577	2,547	2,550
TIGRfam clusters	1345	1362	1355	1369	1369	1372	1365	1362	1372

*For the full table refer to the supporting material (Supplementary File 1). TEIC, Technological Educational Institute of Crete Bacterial collection; CFBP, International Center for Microbial Resources, French Collection for Plant-associated Bacteria, INRA, Angers, France; NCPPB, National Collection of Plant Pathogenic Bacteria, Fera, York, U.K. Isolate names followed by the letter “T” are type strains*.

We used CheckM to assess the completeness and the overall quality of the assemblies (Parks et al., 2015). CheckM was utilized to measure percentage of ambiguous bases, coding density, completeness and contamination (Table 3). We determined that all assemblies were of good quality by assessing them against the thresholds described in Parks et al. (2015). The coding densities of the genomes are within the normal range of 85–90% in bacterial genomes (Ussery et al., 2009).

### General genome features

The basic subsystems of the sequenced genomes were predicted with the RAST server (Overbeek et al., 2014) and the IMG automated annotation pipeline (Markowitz et al., 2014). In particular, we identified differences in proteins predicted to participate in virulence, disease and defense; the number of genes from these families ranged from 106 in strain *Pmed* TEIC1022–127 in strain *Pmed* CFBP5447T (Table 4). However, it is unclear whether these differences represented true differences between strains or if they were due to low quality draft genomes and errors in their assembly.

**Table 4:** **Organismal subsystem prediction overviews estimated by the RAST server**.

**Protein family\Strains (No of total proteins)**	**NCPPB2445T (3957)**	**CFBP5404 (3961)**	**CFBP5403 (3920)**	**CFBP5454 (3993)**	**TEIC1148 (3947)**	**CFBP5447T (4146)**	**CFBP5444 (3985)**	**TEIC1022 (3938)**	**TEIC1105 (3977)**
Cofactors, Vitamins, Prosthetic Groups, and Pigments	347	349	359	351	352	371	348	355	348
Cell, Wall, and Capsule	194	184	206	203	197	197	184	184	184
Virulence, Disease, and Defense	117	107	123	120	123	127	107	106	107
Potassium metabolism	31	32	31	31	31	32	32	32	32
Photosynthesis	0	0	0	0	0	0	0	0	0
Miscellaneous	51	55	51	51	51	55	55	54	55
Phages, Prophages, and Transposable elements	14	27	23	27	12	30	35	28	30
Membrane Transport	212	198	209	216	210	193	200	185	200
Iron acquisition and metabolism	28	30	31	28	28	32	30	30	30
RNA Metabolism	188	188	183	185	184	193	190	191	190
Nucleosides and Nucleotides	127	127	124	128	128	129	127	127	127
Protein Metabolism	272	252	246	248	245	270	250	255	250
Cell division and Cell cycle	34	35	35	34	34	37	35	35	35
Motility and Chemotaxis	130	156	135	130	131	130	157	129	157
Regulation and Cell signaling	117	116	120	116	121	120	117	119	116
Secondary Metabolism	6	6	6	6	6	6	6	6	6
DNA Metabolism	122	126	117	145	131	163	125	125	125
Regulons	8	9	9	9	8	10	8	9	8
Fatty Acids, Lipids, and Isoprenoids	216	218	211	217	212	228	218	230	218
Nitrogen Metabolism	95	85	101	96	95	87	86	87	86
Dormancy and Sporulation	4	5	4	5	5	5	5	5	5
Respiration	138	134	142	144	141	143	135	134	135
Stress Response	193	185	188	193	203	196	188	190	187
Metabolism of Aromatic Compounds	130	130	136	122	126	138	131	134	131
Amino Acids and Derivatives	614	619	597	612	610	631	620	623	619
Sulfur Metabolism	54	75	53	49	49	73	75	75	75
Phosphorus Metabolism	42	33	44	43	44	35	35	34	35
Carbohydrates	473	480	436	484	470	515	486	486	486

*TEIC, Technological Educational Institute of Crete Bacterial collection; CFBP, International Center for Microbial Resources, French Collection for Plant-associated Bacteria, INRA, Angers, France; NCPPB, National Collection of Plant Pathogenic Bacteria, Fera, York, U.K. Isolate names followed by the letter “T” are type strains*.

In addition, we scanned the *Pcor* and *Pmed* genomes for the presence of genes necessary for the biosynthesis of ethylene and auxin. We queried the genomes with the gene coding for ethylene-forming enzyme (*efe*) to assess their capability for ethylene biosynthesis. Similarly, we scanned for the presence of the auxin biosynthesis genes coding for indole-3-acetamide synthase (*iaaM*), indole-3-acetamide hydrolase (*iaaH*) and indoleacetate-lysine ligase (*iaaL*). No significant similarities were found.

### Two non-fluorescent phytopathogenic pseudomonads in the *P. fluorescens* clade

**Figure 2:**
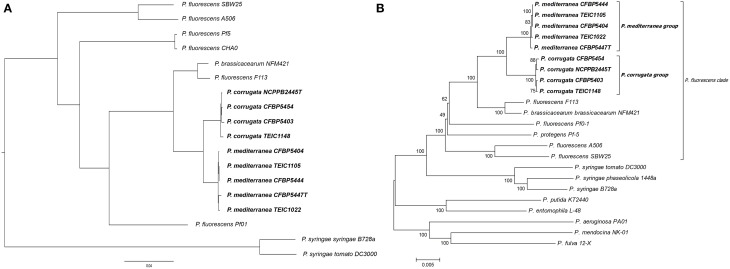
**(A)** Phylogenetic tree of *Pseudomonas* species. Prefixes *Pcor, Pmed, Pbra*, and *Pflu* correspond to species *Pseudomonas corrugata, P. mediterranea, P. brassicacearum*, and *P. fluorescens*, respectively. All nodes had 100% bootstrap support, except the one separating *Pmed* CFBP5447 and *Pmed* TEIC1022, with 98% bootstrap support. The tree was generated using whole genome alignment of orthologous proteins following the approach described in Material and Methods, and was drawn using FigTree (http://tree.bio.ed.ac.uk/software/figtree/). **(B**) Phylogenetic tree of the examined *Pseudomonas* strains based on the concatenated partial sequences of *fruK, gltA, gyrB, mutL, rpoB, rpoD*. Isolate names followed by the letter “T” are type strains. TEIC strains came from the Technological Educational Institute of Crete Collection. CFBP strains came from the French Collection of Plant-associated Bacteria, part of the International Center for Microbial Resources. The evolutionary history was inferred using the neighbor-joining method (Saitou and Nei, 1987). The bootstrap consensus tree was inferred from 1500 replicates (Felsenstein, 1985) and is shown next to the branches. The tree is drawn to scale, with branch lengths in the same units as those of the evolutionary distances used to infer the phylogenetic tree. The evolutionary distances were computed using the maximum composite likelihood method (Tamura et al., 2004) and are in the units of the number of base substitutions per site. The analysis involved 23 nucleotide sequences. All ambiguous positions were removed for each sequence pair. There were a total of 12,292 positions in the final data set. Evolutionary analyses were conducted in MEGA 6 (Tamura et al., 2013).

Based on previous 16S rDNA sequence analysis, both *Pcor* and *Pmed* have been included in the *P. fluorescens* group, while the closest species, in terms of genetic distance, has been reported to be *Pseudomonas brassicacearum* (Anzai et al., 2000; Catara et al., 2002). Here, we generated a whole genome phylogenetic tree from all nine *Pcor* and *Pmed* strains, in addition to the closely related *Pseudomonas* species *P. brassicacearum* subsp. *brassicacearum* NFM421, *P. fluorescens* A506, *P. fluorescens* CHA0, *P. fluorescens* F113, *P. fluorescens* Pf0-1, *P. fluorescens* Pf-5, *P. fluorescens* SBW25, and finally, as out-groups, *P. syringae* pv. *syringae* B728a and *P. syringae* pv. *tomato* DC3000 (Figure 2A). We identified 3826 ortholog families producing, after concatenation, a whole alignment with 1,218,944 columns. All nodes of the phylogenetic tree had 100% bootstrap support, except the node separating strains *Pmed* CFBP5447 and TEIC1022, which had 98% bootstrap support.

Because of the lack of whole genomic sequences for all *Pseudomonas* species, as well as the poor assembly quality of some analyzed genomes, we also used MLSA for six independent loci, to determine the phylogenetic relationships of the nine strains for which genomes were assembled. The selected loci derived from sequences spanning the coding regions of *fruK, gltA, gyrB, mutL, rpoB*, and *rpoD*. Beyond the *Pcor* and *Pmed* strains sequenced in this study, several other strains belonging to different phylogenetic clades were selected. The phylogenetic analysis showed that the *Pcor* and *Pmed* strains cluster in distinct monophyletic clades within the larger *P. fluorescens* clade, clearly differentiated from *P. brassicacearum* subsp. *brassicacearum* NFM421T and *P. fluorescens* F113 (Figure 2B). *P. brassicacearum* has been described as a major root-associated bacterium of *Arabidopsis thaliana* and *Brassica napus*, which may co-exist with *Pcor* and *Pmed* in agricultural niches (Ortet et al., 2011). *P. fluorescens* F113 is a plant growth-promoting rhizobacterium that has biocontrol activity against fungal plant pathogens; it was recently transferred to the *P. brassicacearum* group (Redondo-Nieto et al., 2013). Our phylogenetic results are congruent with MLSA data from Mulet et al. (2010), which grouped *Pcor* in the *P. fluorescens* lineage along with *Pmed, P. kilonensis, P. thivervalensis*, and *P. brassicacearum* (called the *P. corrugata* subgroup). This is also in accordance with earlier MLSA data by Trantas et al. (2015), which described the genetic relatedness of *Pcor* and *Pmed* to *P. brassicacearum* subsp. *brassicacearum* and *P. fluorescens*. Furthermore, in a whole genome based phylogeny, the *P. fluorescens* strain F113 was grouped with *P. brassicacearum* strain Q8r1-96 and the *P. fluorescens* strains Wood1R and Q2-87 (Redondo-Nieto et al., 2013). All cited species and strains in this *P. corrugata* subgroup, whose taxonomic position need to be clarified, were isolated from the rhizosphere or from agricultural soils and mostly studied as bio-control strains of soil-borne plant diseases. Notably, *Pcor* and *Pmed* are the only non-fluorescent and the only plant pathogenic species placed in this group, to date.

### The core and the pan-genome of *P. corrugata* and *P. mediterranea* species

**Figure 3:**
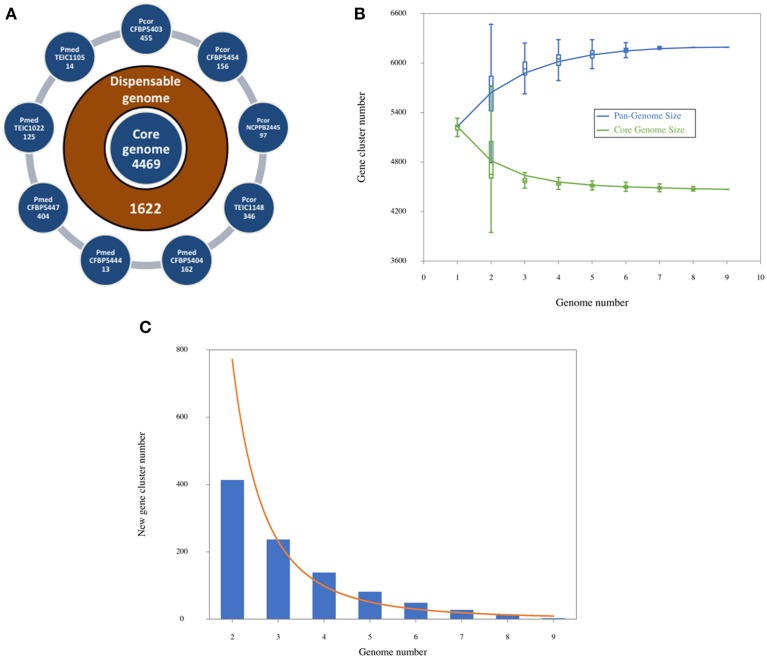
**(A)** Numbers of shared and specific genes between *Pseudomonas corrugata* and *P. mediterranea* based on clusters. External circles show singletons of each genome. The intermediate circle shows the number of dispensable genes, and the inner one shows the core genome size, given by the number of clusters with at least one gene of each genome. **(B)** Core-genome and pan-genome sizes according to the number of genomes considered in the dataset. For each k in X axis, all possible combinations of k genomes (among 9 of this study) are taken and, for each one of these combinations, pan and core numbers are plotted. **(C)** Number of new genes observed upon adding a new genome at each step in the dataset.

We determined the number of shared and strain specific genes based on the clusters found by OrthoMCL (Li et al., 2003), taking protein-coding genes from all nine *Pcor* and *Pmed* as input (Figure 3A). Each external circle shows the number of specific (singleton) genes of each genome. The core genome size is 4469 genes (the number of families with at least one gene in each genome) and the dispensable genome size (sometimes called accessory genome) is 1622 genes. Core-genome and pan-genome rarefaction curves are shown in Figure 3B. The core genome size of the nine genomes presented here is larger than the core genome of the *P. fluorescens* clade earlier reported (Loper et al., 2012), exemplifying the heterogeneity of the clade. On the other hand, the new gene curve (Figure 3C) reached a plateau relatively early, which suggests that there is respective similarity between the nine sequenced strains.

### Proteome comparison

**Figure 4:**
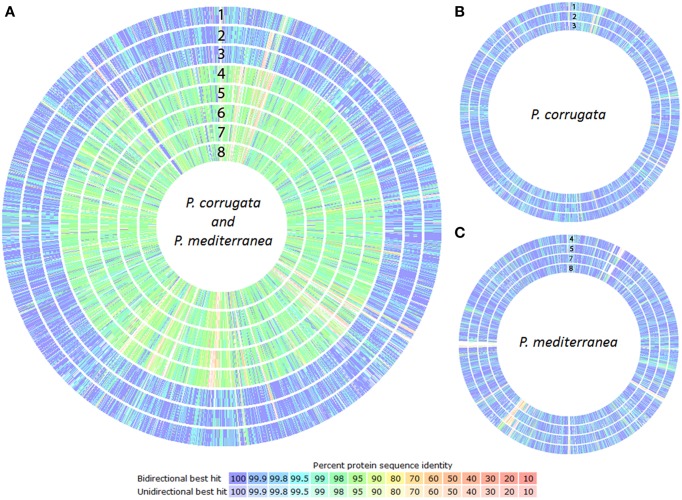
**Proteome comparison of the *Pseudomonas corrugata* (*Pcor*) and *P. mediterranea* (*Pmed*) strains based on RAST genome annotation**. Each proteome is presented as a closed circle with similarity for each protein against the reference genome designated by colored lines. **(A)** *Pcor* and *Pmed* interspecific proteome comparison against *Pcor* NCPPB2445 type strain, **(B)** *Pcor* intraspecific proteome comparison against *Pcor* NCPPB2445 type strain, **(C)** *Pmed* intraspecific proteome comparison against *Pmed* CFBP5447 type strain. 1, CFBP5403; 2, CFBP5454; 3, TEIC1148; 4, CFBP5404; 5, CFBP5444; 6, CFBP5447T; 7, TEIC1022, and 8, TEIC1105. The white gaps and the low similarity areas in intraspecies proteome comparisons correspond to phage genome integrations.

According to the phylogenetic trees constructed either with an MLSA or a whole-genome approach, the *Pcor* and *Pmed* species were identified to be closely related, and both had similar evolutionary distance from strains of *P. fluorescens* and *P. brassicacearum*. The proteomes of the nine sequenced strains were compared against the proteome of *P. brassicacearum* subsp. *brassicacearum* NFM421 (Supplementary File 1), using the RAST proteome comparison pipeline. Proteome similarities within both the *Pcor* and *Pmed* sequenced strains (Figure 4A), as well as intraspecific similarities between *Pcor* (Figure 4B) and *Pmed* (Figure 4C) strains are present.

### Protein secretion and translocation systems

Protein and DNA translocation into neighboring cells and secretion to the extracellular environment is achieved by several secretion/translocation systems in Gram negative bacteria. These secretion systems have been classified into six types, from the type I secretion system through to the type VI secretion system (T1SS-T6SS) (Tampakaki et al., 2010).

We screened the genomes of *Pcor* and *Pmed* type strains for the evidence of these secretion/translocation systems, using the gene clusters of *P. fluorescens* F113 as sequence queries. Our data revealed that both *Pcor* and *Pmed* genomes encode a wide variety of secretion systems, including six T1SSs, two T2SSs, one T4SS (detected only partially in *Pcor* strains), five T5aSSs, three T5bSS, one T5dSS (which is detected only in *Pmed* strains) and three T6SSs (Table 5). Surprisingly, no T3SS-encoding locus was detected in any of the examined strains.

**Table 5:** **The full repertoire of protein secretion/translocation systems of *Pseudomonas corrugata* (*Pcor*) and *P. mediterranea* (*Pmed*) type strains**.

**Secretion system**		***P. corrugata* NCPPB2445T**	***P. mediterranea* CFBP5447T**	***P. fluorescens* F113**
T1SS		PcorDraft_00553-00551	PmedDraft_01363-01365	PSF113_0209-0211
		PcorDraft_00252	PmedDraft_02514	PSF113_0530
		PcorDraft_00971-00969	PmedDraft_00943-00941	PSF113_1508-1510
		PcorDraft_05030-05032	PmedDraft_04444-04442	PSF113_2734-2736
		PcorDraft_00188-00190	PmedDraft_04408-04410	PSF113_2945-2947
		PcorDraft_04243-04245	PmedDraft_05697-05699	PSF113_3005-3007
		PcorDraft_03958-03956	PmedDraft_00478-00480	PSF113_3303-3305
T2SS	Xcp	PcorDraft_04315-04305	PmedDraft_01591-01607	PSF113_0437-0447
	Putative substrates	PcorDraft_04316 PcorDraft_03149 PcorDraft_03269 PcorDraft_02349	PmedDraft_01594 PmedDraft_05894 PmedDraft_04897 PmedDraft_05316 PmedDraft_00632	PSF113_0435, PSF113_0610, PSF113_2337, PSF113_3279
	Hxc	PcorDraft_00325-00347	PmedDraft_04580-04583	PSF113_3690-3700
T3SS	SPI-I	not detected in *Pcor* strains	not detected in *Pmed* strains	PSF113_1778-1801
	Hrp1	not detected in *Pcor* strains	not detected in *Pmed* strains	PSF113_5585-5610
T4SS	GI-like	not detected in strain CFBP5447 (partially only in TEIC1148 and CFBP5403)	PmedDraft_00600-00584	PSF113_3314-3334
T5aSS		PcorDraft_02455	PmedDraft_03164	PSF113_2779
		PcorDraft_02456	PmedDraft_03163	PSF113_2780
		PcorDraft_04540	PmedDraft_05328	PSF113_4399
		PcorDraft_04609	PmedDraft_01659	PSF113_5339
		PcorDraft_00187	PmedDraft_00248	PSF113_5848
T5bSS		PcorDraft_03404-03405	PmedDraft_00300-00301	PSF113_0792-0793
		PcorDraft_02193-02192	PmedDraft_00959-00960	PSF113_1489-1490
T5dSS		not detected in Pcor strains	PmedDraft_00929	PSF113_1517
T6SS	I	PcorDraft_01669-01644	PmedDraft_03358-03331	PSF113_5785-5808
	II	PcorDraft_04761-04784	PmedDraft_05238-05225	PSF113_2407-2422
	III	PcorDraft_05211-05196	not detected in strains CFBP5447 and TEIC1022	PSF113_5815-5833
	Putative substrates	PcorDraft_05064 (hcp)	PmedDraft_03683 (hcp)	
		PcorDraft_00287 (vgrG)	PmedDraft_02478 (vgrG)	
		PcorDraft_01038 (vgrG)	PmedDraft_03454 (vgrG)	
		PcorDraft_00092 (vgrG)	PmedDraft_04237 (vgrG)	
		PcorDraft_04873 (vgrG)	PmedDraft_03810 (vgrG)	
		PcorDraft_03798 (vgrG)	PmedDraft_04962 (vgrG)	

*TEIC, Technological Educational Institute of Crete Bacterial collection; CFBP, International Center for Microbial Resources, French Collection for Plant-associated Bacteria, INRA, Angers, France; NCPPB, National Collection of Plant Pathogenic Bacteria, Fera, York, U.K. Isolate names followed by the letter “T” are type strains. Xcp, extracellular protein; Hpx, homolog of Xcp; SPI-I and SPI-II, T3SS homologs*.

### Type II secretion system

The type II secretion system (T2SS) is used by Gram negative bacteria to secrete proteins into the extracellular environment, and it serves as a major virulence mechanism in several bacterial species. It is well-conserved and is composed of a set of 11 to 12 proteins (Whitchurch et al., 2004). *Pseudomonas* T2SSs could be divided in two main clusters, named Xcp (extracellular protein) and Hpx (homolog to Xcp) (Redondo-Nieto et al., 2013). Similar to the closely related species *P. fluorescens* F113, two T2SS-encoded loci, one related to Xcp cluster T2SSs and the other related to Hxc cluster T2SSs, are present in the genome of both *Pcor* and *Pmed* species (Table 5). The genetic organization of the *Pcor* and *Pmed* Hxc cluster is highly similar to the Hxc clusters of *P. fluorescens* F113 and *P. aeruginosa* PAO1 (Ball et al., 2002; Redondo-Nieto et al., 2013). Furthermore, at least five predicted *Pcor* and *Pmed* proteins could potentially be Xcp substrates (Table 5), based on reported similarities with known Xcp effectors.

### *P. corrugata* and *P. mediterranea* lack a T3SS

The type III secretion system (T3SS) is a bacterial molecular nano-syringe related to the bacterial flagellum and is required for the translocation of virulence proteins. It is composed of approximately 25 proteins (Tampakaki et al., 2010; Skandalis et al., 2012), and has evolved into seven different families: Ysc, Hrp1, Hrp2, SPI-1, SPI-2, *Rhizobiaceae* and *Chlamydia* (Pallen et al., 2005; Troisfontaines and Cornelis, 2005).

An in-depth investigation of the proteomes of other *Pseudomonas* species revealed 37, 32, 47, and 57 T3SS-associated protein families in *P. brassicacearum, P. fluorescens, P. syringae* pv. *syringae*, and in *P. syringae* pv. *tomato*, respectively.

The predicted coding sequences from *Pcor* and *Pmed* genomes were translated and screened with several Hrp/Hrc homologs from *P. syringae, P. brassicacearum, Xanthomonas*, and *Erwinia* T3SSs, including a second rhizobial type T3SS cluster, which has been identified in several *P. syringae* strains (Gazi et al., 2012). From the 4,469 gene families of *Pcor* and *Pmed* species, only five that belong to the bacterial flagellum machinery revealed similarity to T3SS.

We used the amino acid sequences of all type III effector proteins (T3Es) obtained from the PPI Home web page (http://www.pseudomonas-syringae.org/) to screen for T3Es in the predicted proteomes of *Pcor* and *Pmed*. We identified only one T3E-like protein, similar to HopPmaJ (HopJ), conserved in all examined strains. BLASTP revealed that this protein is conserved in all pseudomonads in *P. fluorescens* group including many non-pathogenic strains. However, as far as we are aware there is not confirmation for the T3SS-dependent secretion of this particular protein.

The absence of a *Hrp* gene cluster from the genomes of *Pcor* and *Pmed* strains is a unique feature of these pathogens, since the majority of the known plant, insect and animal pathogenic pseudomonads rely on the presence of a functional T3SS to manipulate immunity and to colonize their hosts (Tampakaki et al., 2004). However, *P. aeruginosa* isolates that lack a T3SS have become established in hosts by relying on the initial infection and suppression of host immunity by T3SS-positive isolates (Czechowska et al., 2014). The absence of genes encoding a T3SS in *Pcor* and *Pmed* strains, in combination with the induction of an HR-like response in *Nicotiana* plants, potentially indicates the presence of additional virulence mechanisms, which probably include the secretion of apoplastic effectors. These theories remain to be investigated and our work provides the basis for future studies on the characterization of *Pcor* and *Pmed* virulence strategies.

Similar virulence strategies have been described for some Gram positive plant pathogens (Eichenlaub and Gartemann, 2011). For example, *Clavibacter michiganensis* relies on small secreted proteins (e.g., Chp7: CMS_2989 and Pat1: pCM2_0054) to confer virulence. We used the amino-acid sequences of these proteins as baits for screening the *Pcor* and *Pmed* predicted proteomes. No similar protein-coding genes were found in either species.

### Type VI secretion system

The T6SS was initially identified as a protein secretion apparatus involved in virulence of *Vibrio cholerae* on *Dictyostelium* (Mougous et al., 2006) and *P. aeruginosa* on mouse models (Pukatzki et al., 2006). Furthermore, gene clusters coding for the type VI secretion system (T6SS) are present in the majority of plant pathogenic and symbiotic Gram negative bacterial genomes (Sarris et al., 2012), which indicates its importance in the life cycles of these species. Although the exact role of the T6SS in plant colonization is not clear, it is noteworthy that there are multiple T6SS clusters in single strains.

**Figure 5:**
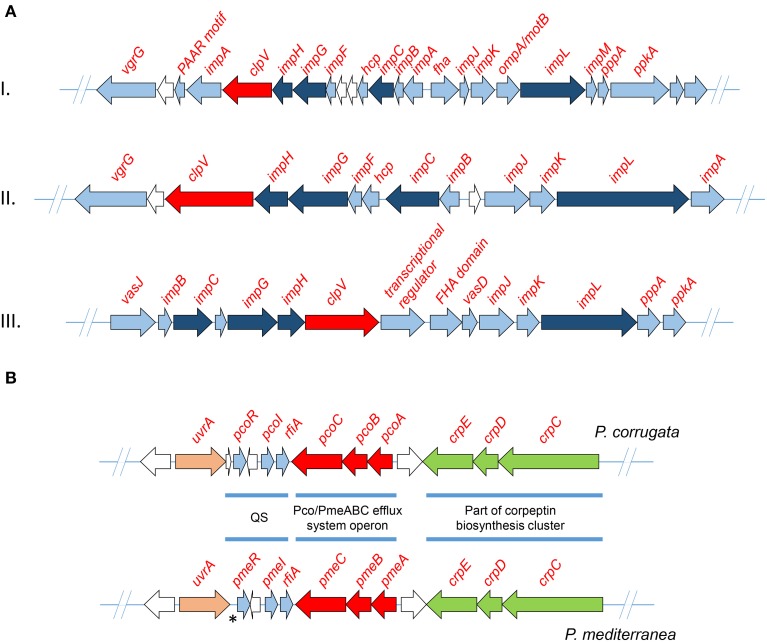
**(A)** Maps of the three detected T6SS clusters of *Pseudomonas corrugata* and *P. mediterranea* strains. Arrows indicate detected ORFs and their direction shows the direction of transcription. All ORFs annotated as type VI secretion system associated proteins are colored light blue. Those which designate the T6SS phylogeny are colored dark blue. The ClpV protein, which was used to mine the T6SS from each genome, is colored red. White arrows indicate ORFs encoding hypothetical, uncharacterized proteins. **(B)** The map of *P. corrugata* and *P. mediterranea* consensus quorum sensing gene clusters. The asterisk indicates the locus of *P. mediterranea* that lacks an ORF when compared to the genome of *P. corrugata*. QS, quorum sensing.

Three phylogenetically distinct clusters coding for T6SSs have been characterized in the genome of various *P. syringae* pathovars (Sarris et al., 2010, 2012). In order to identify gene clusters coding for T6SS components of the assembled genomes, all the *Pcor* and *Pmed* predicted proteomes were screened using the T6SS core components ImpB, ImpC and the ClpV1 ATPase of *P. syringae* as sequence queries. BLASTP analysis of the nine predicted proteomes was used to reveal the presence of three independent gene clusters that were extracted from each genome (Table 5). Several genes coding for VgrG (Valine-Glycine Repeat Protein G) proteins were found randomly distributed in the genome of both species. This is a common feature of *Pseudomonas* species (Sarris et al., 2010, 2012; Sarris and Scoulica, 2011). Nucleotide alignments of the three T6SS clusters revealed a high degree of similarity between the nine sequenced strains of *Pcor* and *Pmed*, and the *P. brassicacearum* strains. Interestingly, the T6SS-III locus was not identified in *Pmed* strains TEIC1022 and CFBP5447 or in *P. brassicacearum* NFM421, but the cluster is present in the genome of *P. fluorescens* F113. The three phylogenetically-distinct consensus T6SS loci are schematically presented in Figure 5A.

**Figure 6:**
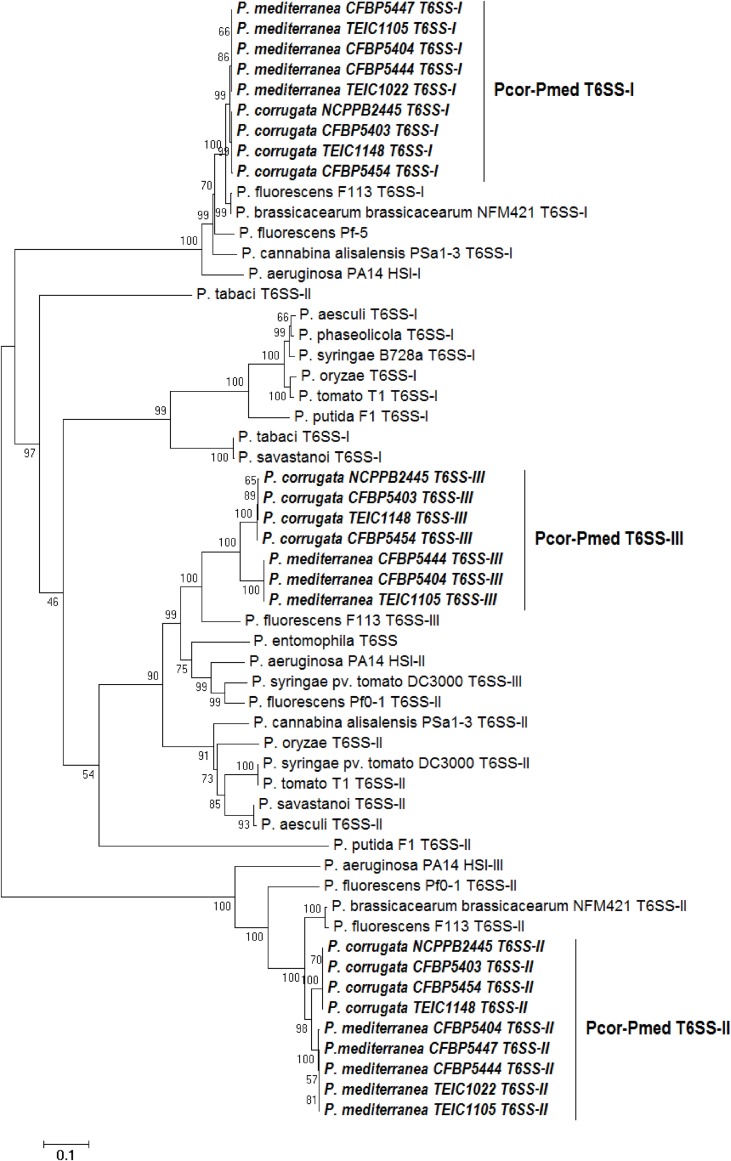
**Distance tree of T6SSs of various pseudomonads based on the sequence of four T6SS core proteins (ImpC, ImpG, ImpH, and ImpL)**. The evolutionary history was inferred using the Neighbor-Joining method (Saitou and Nei, 1987). The bootstrap consensus tree inferred from 1500 replicates is taken to represent the evolutionary history of the proteins analyzed (Felsenstein, 1985). The tree is drawn to scale, with branch lengths in the same units as those of the evolutionary distances used to infer the phylogenetic tree. The evolutionary distances were computed using the Poisson correction method (Zuckerkandl and Pauling, 1965) and are in theunits of the number of amino acid substitutions per site. The analysis involved 59 amino acid sequences. All positions containing gaps and missing data were eliminated. There were a total of 829 positions in the final dataset. Evolutionary analyses were conducted using MEGA6 (Tamura et al., 2013). “*Pcor*” stands for *Pseudomonas corrugata* while “*Pmed”* stands for *P. mediterranea*. “TEIC” strains came from the Technological Educational Institute of Crete Collection. CFBP strains came from the French Collection of Plant-associated Bacteria, part of the International Center for Microbial Resources.

Phylogenetic analysis of various *Pseudomonas* species was performed using concatenated protein sequences of four highly conserved T6SS core proteins (ImpC, ImpG, ImpH, ImpL) (Figure 6). The consensus phylogenetic tree obtained indicates that pseudomonad T6SSs are scattered into three main clusters (Figure 6), and this matches what has been previously shown (Sarris et al., 2012).

The T6SS clades from *Pcor* and *Pmed* tend to cluster with sequences from *P. fluorescens* F113 and *P. brassicacearum* NFM421. Our analysis revealed that the T6SS-II clusters of *Pcor* and *Pmed* are highly related to the T6SS-III of the opportunistic human pathogen *P. aeruginosa* (Figure 6). In addition, the grouping of *Pcor* and *Pmed* indicates minor divergence of the T6SS between the two species, since no mixing among the *Pcor* and *Pmed* strains was observed. The divergence was higher for the T6SS-II and T6SS-III clusters than for the T6SS-I cluster.

### Other virulence factors

#### Quorum sensing

Highly similar AHL-QS systems were identified in *Pcor* strain CFBP5454 and *Pmed* strain CFBP5447; these LuxI/R QS systems were designated PcoI/R and PmeI/R, respectively (Licciardello et al., 2007, 2012). As described earlier, the AHL-QS system and more specifically the PcoR and PmeR, as well as the RfiA regulators of both species, are required for full virulence in tomato (Licciardello et al., 2009, 2012; Strano et al., 2014). Analysis of all examined genomes revealed that all strains have genes of only one canonical paired LuxI/R system (Figure 5B). The QS gene topology was conserved in both *Pcor* and *Pmed* strains, with the *luxI/luxR* genes oriented in the same direction and separated by a divergently oriented ORF, which codes for a putative homoserine/threonine efflux protein. All strains have the *rfiA* gene coding for a transcriptional regulator downstream of, and in the same orientation as, *luxI*. Licciardello et al. demonstrated that the two genes are co-transcribed in both species (Licciardello et al., 2009, 2012).

The LuxI and LuxR protein sequences are highly conserved within *Pcor* (between 99 and 100%) and *Pmed* species (100%) and also between the two species (similarities >84% and >95%, respectively). A significant difference was observed between the promoter regions of *pcoR* and *pmeR*; the promoter region of *Pmed* strains is 31 bp longer than that of *Pcor*. It is not known whether this property may have any effects on the regulation of *pmeR* compared to *pcoR*, but it could account for some of the differences observed between the two species.

The regulatory gene, *rfiA*, was located at the right border of the PcoI/PmeI gene in all strains. RfiA contains a C-terminal helix-turn-helix (HTH) DNA-binding motif, which is characteristic of the LuxR family of bacterial regulatory proteins, but lacks the auto-inducer-binding domain found in the quorum-sensing-associated LuxR regulators (Licciardello et al., 2009). RfiA showed a similarity of 100% within species and >94% between species. These kinds of LuxR-type regulators have been demonstrated to be involved in the biosynthesis of a number of cyclic lipopeptides in several pseudomonads (reviewed in Raaijmakers et al., 2010).

#### *P. corrugata* and *P. mediterranea* harbor a large arsenal of secondary metabolites

Many plant associated *Pseudomonas* produce secondary metabolite cyclic lipopeptides (CLPs) that are composed of a fatty acid tail, linked to a short oligo-peptide which is cyclized to form a lactone ring between two amino acids in the peptide chain. They possess surfactant, antimicrobial, anti-predation, and cytotoxic properties (Raaijmakers et al., 2006, 2010). Based on the length and composition of the fatty acid tail as well as the number, type, and configuration of the amino acids in the peptide moiety, CLPs have been classified into six groups. Biosynthesis of CLPs is directed by non-ribosomal peptide synthetases (NRPS), which are multi-enzyme complexes with a modular arrangement. Each module consist of several domains for condensation (C), thiolation (T) and aminoacyl adenylation (A), responsible for incorporation, recognition and activation of each amino acid unit into the growing peptide (Raaijmakers et al., 2006). Genes that code for these large multi-modular enzymes can span up to 75 kb of DNA. This repetitive structure is difficult to assemble with next generation sequencing approaches; such large enzymes may be split and span several contigs. This is the case for *Pcor* and *Pmed* genomes, for which the software pipeline antiSMASH (Blin et al., 2013) detected corpeptin NRPS spanning several contigs.

*Pcor* and *Pmed* strains produce phytotoxic and antimicrobial cationic CLPs (Emanuele et al., 1998; Scaloni et al., 2004; Licciardello et al., 2012). Corpeptin A and corpeptin B, two isoforms consisting of 22 amino acid residues, were identified in the culture of the *Pcor* type strain. Both are phytotoxic against tobacco leaves and possess antimicrobial activity against *Bacillus megaterium* (Emanuele et al., 1998). A strain-dependent production of cormycin, a lipodepsinonapeptide, has been earlier described (Scaloni et al., 2004). This molecule produced a strong *in vitro* inhibition of *B. megaterium* and *Rhodotorula pilimanae* that was higher than that of nonapeptides from *P. syringae*, and also induced chlorosis in tobacco leaves (Scaloni et al., 2004).

*Pcor* and *Pmed* are able to inhibit the *in vitro* growth of various Gram positive and Gram negative bacteria, and phytopathogenic fungi (Catara, 2007). The antimicrobial activity of strains described in this study was tested against two microorganisms: the Gram positive bacterium *Bacillus megaterium* ITM100 and the yeast *Rhodotorula pilimanae* ATTC26432, which are both sensitive to CLPs, and against the Gram negative phytopathogens *P. syringae* pv. *tomato* PVCT28.3.1 and *X. campestris* pv. *campestris* PVCT 62.4. All *Pcor* and *Pmed* strains displayed activity that inhibited the growth of *R. pilimanae*. Only the five R-phenotype strains were able to inhibit the growth of *B. megaterium* and *P. syringae* pv. *tomato* (Figure 1D and Table 2). Cormycin and corpeptins are both produced in cultures which displayed antimicrobial activity, so we therefore decided to test the culture filtrates of four strains grown in CLP-inducing conditions; two for *Pcor* (CFBP5454, CFBP5403) and two for *Pmed* strains respectively (CFBP5447, CFBP5444) (Surico et al., 1988). The culture filtrates of three strains sharing the R-phenotype showed antimicrobial activity against both *R. pilimanae* and *B. megaterium*, whereas *Pmed* CFBP5444 S-type strain culture filtrate did not display antagonistic activity (Figure 1D). This correlates with the previous observation that culture filtrates of *Pcor* strain CFBP5454 and *Pmed* CFBP5447 mutants unable to produce these CLPs lose the ability to inhibit the *in vitro* growth of the two bio-indicator microorganisms (Licciardello et al., 2009, 2012). Moreover, a null mutant strain for the QS transcriptional regulator PcoR, which is impaired in CLP production, is still able to antagonize fungal and bacterial plant pathogens in dual culture assays (Licciardello et al., 2007). Thus, we propose that the residual antifungal activity against *R. pilimanae* is due at least partially to another secreted antifungal metabolite. These results support the importance of further in-depth studies for the identification of antimicrobial components of both *Pcor* and *Pmed* species for their application against important plant pathogens.

#### Antimicrobial cluster mining

We used the software pipeline antiSMASH (Blin et al., 2013) for the automated identification of secondary metabolite biosynthesis clusters in each genome. The analysis identified, in all genomes, a number of non-ribosomal peptide synthetases (NRPS) clusters, a gene cluster related to bacteriocin biosynthesis, a type I PKS cluster a siderophore and an arylpolyene gene cluster. A lantipeptide gene cluster was exclusively detected in *Pmed* genomes (Supplementary File 2).

In the *Pcor* CFBP5454 genome, which has a relatively low number of scaffolds, we identified at least 10 scaffolds with genes similar to those for syringomycin and syringopeptin biosynthesis, secretion and regulation in *P. syringae* pv. *syringae* strains. In particular, we found NRPSs putatively involved in cormycin and corpeptin production and their secretion. One of the antiSMASH clusters matches with the GenBank sequence KF192265. This cluster also includes genes for the PcoI/PcoR AHL-QS system, the transcriptional regulator gene RfiA and CrpCDE corpeptin biosynthetic cluster coding for an approximately 6000 bp DNA segment that encodes two modules of a NRPS (*crpC*) and an ABC transporter responsible for corpeptin secretion (*crpDE*) (Strano et al., 2014). The CrpDE efflux system is similar to the PseEF efflux system of *P. syringae* pv. *syringae* strain B301D, which is involved in the export of syringomycin and syringopeptin (Cho and Kang, 2012). The scaffolds containing the QS system have the same topology in all *Pcor* and *Pmed* strains but the sequence of the corpeptin synthetase gene C (*crpC*) is always prematurely interrupted by the end of a contig. The analysis of NRPS clusters in the other strains supported the presence of the same cormycin and corpeptin biosynthesis, secretion and regulatory apparatus for all strains.

BLAST searches of the other NRPS genes mined by antiSMASH suggest that three other different NRPS clusters are present in all strains. One of them is putatively involved in the production of the lipopeptide siderophore corrugatin (see Siderophores paragraph below). Thus, more bioactive metabolites can be produced by the strains by means of the other two NRPS clusters and by a conserved type I PKS cluster which was not detected in other *Pseudomonas* species deposited in GenBank and IMG databases. Best similarities (approximately 40%) were detected by BLAST within the genomes of the symbiotic bacterium *Rhizobium sullae* wsm1592 and *Mesorhizobium loti* MAFF303099 plasmid pMLa.

In addition, an aryl-polyene (APE) gene cluster was detected in all strains Recently APE gene clusters were found widely but discontinuously distributed among Gram-negative bacteria (Cimermancic et al., 2014). Aryl polyenes are responsible for yellow pigmentation such as the brominated aryl- polyenes xanthomonadins of *Xanthomonas* species (Cimermancic et al., 2014). Lantipeptides, which are potentially bioactive metabolites, are estimated to be produced only by *Pmed* strains. Lantipeptides are ribosomal synthesized and post-translational modified peptides, formerly called lantibiotics, characterized by thio-ether cross-links (Knerr and van der Donk, 2012).

We mined each genome by BLAST using the biosynthetic loci of compounds contributing to biological control in other *Pseudomonas* strains such as phenazines, hydrogen cyanide (HCN), pyrrolnitrin, 2,4-diacetylphloroglucinol (DAPG), and pyoluteorin as queries (Loper et al., 2012). Only the hydrogen cyanide gene cluster was found in all *Pcor* and *Pmed* genomes. However, using the Cyantesmo detection card only, six out of the nine strains produced detectable levels of HCN (Table 2). The HCN and the DAPG gene clusters have been reported in strain Q8r1-96 of the closely related *P. brassicacearum* and in *P. fluorescens* strain F113 (Loper et al., 2012; Redondo-Nieto et al., 2013).

#### Mining for bacteriocins

The assembled genomes were queried for genes encoding bacteriocins (Riley and Wertz, 2002), which are antimicrobial peptides produced by bacteria to inhibit the growth of closely related bacterial strains (Hassan et al., 2012). The translated ORFs of the nine genomes under study and the *P. brassicacearum* NFM421 were blasted with antimicrobial peptides from the BAGEL database (Van Heel et al., 2013) and protein hits were recorded (Supplementary File 1). In all *Pmed* strains, a gene for the synthesis of bacteriocin Colicin E was detected. However, no Colicin E biosynthesis genes were detected in the genomes of the *Pcor* strains, although genes for the peptides Carocin D and Zoocin A were detected. In the related *P. brassicacearum* NFM421, no bacteriocin biosynthesis genes were detected, while in the *P. fluorescens* F113 a Carocin D biosynthesis gene was detected.

#### Siderophores

Typically, *Pseudomonas sensu stricto* produces yellow-green fluorescent pigments when grown under iron-limiting conditions. These fluorescent compounds are pyoverdines that act as siderophores (Cornelis and Matthijs, 2002), which are iron chelating compounds secreted to survive during conditions of iron deprivation. As expected, since *Pcor* and *Pmed* are members of the non-fluorescent group of the species, no pyoverdine biosynthesis cluster was found.

However, *Pcor* was found to produce corrugatin, a CLP siderophore (Meyer et al., 2002). The antiSMASH analysis of *Pcor* and *Pmed* genomes revealed the presence of two different gene clusters putatively coding for proteins involved in siderophore biosynthesis or utilization. The first cluster was characterized by the presence of NRPS genes and was split in all strains into two different contigs (Supplemental File 1). The second cluster revealed similarity to the achromobactin biosynthesis cluster. BLASTX analysis with the predicted NRPS sequences showed similarity to a putative siderophore gene cluster in *P. fluorescens* strain SBW25 (Cheng et al., 2013). The SBW25 gene cluster contains five NRPS genes (PFLU3220, 3222–3225) that are predicted to encode an eight-amino acid peptide that resembles ornicorrugatin (similar to corrugatin, but the amino acid 2,4-diaminobutanoic acid is replaced by the amino acid ornithine), a siderophore produced by *P. fluorescens* AF76 (Matthijs et al., 2008). This cluster is also present in *P. brassicacearum* subsp. *brassicaceraum* NFM421 and in *P. fluorescens* F113, the genomes of which are closely related to the *Pcor* and *Pmed* strains.

We discovered a cluster that has high similarity to the achromobactin biosynthesis and utilization cluster of *P. syringae* pv. *syringae* B728a (Psyr2582-2593) and that has been detected in six different *P. syringae* pathovars (Berti and Thomas, 2009). All *Pcor* and *Pmed* strains in this study possess highly conserved genes of this cluster, but lack the entire ABC transporter system encoded by cbrA, cbrB, cbrC (Psyr2590-2592). A comparative analysis with the genetically related bacterial strains *P. brassicacearum* subsp. *brassicaceraum* strain NFM421 and *P. fluorescens* strain F113 showed the same cluster without the transporter system.

## Conclusion

We sequenced the genomes of nine *P. corrugata* and *P. mediterranea* strains in order to undertake an in-depth genome comparison study. The comparison of nine genomes with each other and with other closely related genomes revealed the absence of virulence genes corresponding to the T3SS from the genome of *Pcor* and *Pmed*. These findings raise the question regarding the absence of a T3SS from *Pcor* and *Pmed*. Was this system deleted specifically in these lineages? This may have happened at a much earlier point in the lineage, or the *P. fluorescens* lineages that have a T3SS may have acquired it via horizontal gene transfer (HGT); this second conclusion would mean that the ancestor of *Pcor* and *Pmed* lineages did not have this cluster. Our whole genome phylogeny provides a potential answer to these questions. According to our analysis, the closer relatives to *Pcor* and *Pmed* species (the *P. brassicacearum* strains NFM421, F113 and the *P*. *fluorescens* strains SBW25, A506), possess a T3SS indicating that the loss of the T3SS from *Pcor* and *Pmed* strains occurred specifically in these species. However, further studies are required for the elucidation of the mechanisms of virulence (e.g., through the exploitation of toxins) of these species.

Although the examined *Pcor* and *Pmed* strains were isolated from different geographic areas and hosts, no host or geographical differentiation in their genomes could be identified. Based on these findings, we hypothesize that the two species may have separated by factors other than the selection pressures of host range and geography. Likewise, differentiation of colony morphology, experimental antimicrobial activity and induction of an HR-like response in *N. benthamiana* plants, resulted in intra- instead of inter-species differentiation for all *Pcor* and *Pmed* strains used in this study. Furthermore, analysis of *Pcor* and *Pmed* genomes showed three genomic clusters coding for T6SSs (T6SS-I, T6SS-II and T6SS-III). The number of T6SS clusters per genome reflects the importance of this secretion system to the life cycle of *Pcor* and *Pmed* species. Interestingly, the first two T6SS clusters are syntenically and phylogenetically related to the *P. aeruginosa* HSI-I and HSI-III. However, the potential contribution of the T6SS to pathogenicity of *Pcor* and *Pmed* species remains to be investigated; this will be of great interest in light of the absence of a functional T3SS in both species.

*Pcor* and *Pmed* are particularly interesting pathogens because they can cause disease on a large number of phylogenetically divergent hosts, including several economically important crop species. This indicates that both *Pcor* and *Pmed* are able to manipulate host immunity without using any known virulence effector protein (e.g., type III effectors). The phylogenetic relationship with saprophyte species *P. fluorescens* and *P. brassicacearum* places *Pcor* and *Pmed* as intermediates between saprophytic and plant pathogenic lifestyles. To date, *Pcor* and *Pmed* have displayed peculiar and unique features. Now that we have analyzed and compared their genomes, future investigations have resources with the exciting potential to reveal the presence of additional virulence mechanisms in the genomes of *Pcor* and *Pmed* and the elucidation of the nature and role of these mechanisms during host colonization. Our study provides the basis for such investigations, and not only gives mechanistic insights into the molecular interactions underlying alternative virulence mechanisms.

## Author contributions

PS and VC conceived the study, and contributed to its design and coordination and helped to draft the manuscript. DG contributed to genome sequencing. ET, GL, NA, KW, DG, VC, and PS contributed to genome assembly and annotation of the genomes. NA contributed to core genome analyses. FV, DG, and JJ contributed materials or analytic tools. VC, CS and DG contributed to antimicrobial and pathogenicity tests. All authors contributed to the writing and editing of the manuscript and approved the final version of it.

### Conflict of interest statement

The authors declare that the research was conducted in the absence of any commercial or financial relationships that could be construed as a potential conflict of interest.
