# Supplementary material for: Comparative genomic analysis of multiple strains of two unusual plant pathogens: Pseudomonas corrugata and Pseudomonas mediterranea
Source: Front Microbiol. 2015 Aug 7;6:811. doi: 10.3389/fmicb.2015.00811 (PMC4528175; doi:10.3389/fmicb.2015.00811)
Supplement: Supplementary file 2 [file DataSheet1.DOCX]

***Supplementary Material***

***Pseudomonas corrugata* and *Pseudomonas mediterranea* two unusual plant pathogens: Comparative genomic analysis of multiple strains**

**Emmanouil A. Trantas^1^, Grazia Licciardello^2^, Nalvo F. Almeida^3^, Kamil Witek^4^, Cinzia P. Strano^5‡^, Zane Duxbury^4^, Filippos Ververidis^1^, Dimitrios E. Goumas^1^, Jonathan D. G. Jones^4^, David S. Guttman^6^, Vittoria Catara^5§^, Panagiotis F. Sarris^1, 4§*^**

^1.^Plant Biochemistry and Biotechnology Laboratory, Department of Agriculture, School of Agriculture and Food Technology, Technological Educational Institute of Crete, Heraklion, Greece.

^2.^ Science and Technology Park of Sicily, Catania, Italy.

^3.^School of Computing, Federal University of Mato Grosso do Sul, Mato Grosso do Sul, Brazil

^4.^ The Sainsbury Laboratory, John Innes Centre, Norwich Research Park, Norwich, UK.

^5.^ Department of Agriculture and Food Science, University of Catania, Catania, Italy.

^6.^ Centre for the Analysis of Genome Evolution & Function, University of Toronto, Ontario, Canada.

*** Correspondence: Dr. Panagiotis F. Sarris, The Sainsbury Laboratory, John Innes Centre, Norwich Research Park, Norwich, UK**

**Panagiotis.Sarris@sainsbury-laboratory.ac.uk**

1. **Supplementary Figures and Tables**

## Supplementary Tables

**Supplementary Table 1.** Full table with genomic statistics.

|  | ***P. corrugata*** | | | | ***P. mediterranea*** | | | | |
| --- | --- | --- | --- | --- | --- | --- | --- | --- | --- |
|  | **CFBP5403** | **CFBP5454** | **NCPPB2445** | **TEIC1148** | **CFBP5404** | **CFBP5444** | **CFBP5447** | **TEIC1022** | **TEIC1105** |
| **Total number of reads** | 11,800,202 | 8,420,692 | 1,519,930 | 8,608,470 | 1,209,534 | 1,394,308 | 24,635,686 | 1,276,402 | 23,866,648 |
| **Average read length** | 35 | 70.6 | 145 | 35 | 145 | 145 | 51 | 145 | 44 |
| **Total bases** | 413,007,070 | 594,525,036 | 220,389,850 | 301,296,450 | 175,382,430 | 202,174,660 | 1,256,357,465 | 185,078,290 | 1,040,233,200 |
| **DNA, total number of bases in contigs** | 6,166,582 | 6,195,615 | 6,084,011 | 6,266,776 | 6,281,542 | 6,298,862 | 6,270,666 | 6,231,327 | 6,299,426 |
| Ambiguous bases | 39,282 | 2,299 | 3,749 | 31,312 | 3,143 | 3,499 | 18 | 11,988 | 1,651 |
| Percentage of ambiguous bases | 0.64 | 0.04 | 0.06 | 0.50 | 0.05 | 0.06 | 0.00 | 0.19 | 0.03 |
| DNA coding number of bases | 5,471,555 | 5,491,990 | 5,391,923 | 5,567,198 | 5,564,235 | 5,572,667 | 5,573,461 | 5,507,149 | 5,573,467 |
| DNA G+C number of bases | 3,706,298 | 3,747,457 | 3,686,460 | 3,763,425 | 3,846,554 | 3,853,935 | 3,862,577 | 3,813,571 | 3,855,401 |
| **Total number of contigs** | 442 | 84 | 104 | 432 | 150 | 89 | 109 | 101 | 91 |
| **Genes total number** | 5,912 | 5,570 | 5,463 | 5,845 | 5,652 | 5,638 | 5,533 | 5,594 | 5,643 |
| Coding density (%) | 87.9 | 88.5 | 88.4 | 88.4 | 88.4 | 88.3 | 88.4 | 88.1 | 88.3 |
| Completeness (%) | 97.02 | 99.69 | 99.68 | 99.14 | 99.69 | 99.68 | 99.69 | 99.45 | 99.69 |
| Contamination (%) | 0.80 | 0.42 | 0.45 | 0.47 | 0.48 | 0.79 | 0.48 | 0.43 | 0.79 |
| Protein coding genes | 5,809 | 5,443 | 5,313 | 5,737 | 5,515 | 5,484 | 5,786 | 5,450 | 5,481 |
| RNA genes | 103 | 127 | 150 | 108 | 137 | 154 | 135 | 144 | 162 |
| rRNA genes | 3 | 3 | 4 | 4 | 4 | 4 | 9 | 5 | 5 |
| 5S rRNA | 1 | 1 | 2 | 2 | 2 | 2 | 3 | 3 | 2 |
| 16S rRNA | 1 | 1 | 1 | 1 | 1 | 1 | 3 | 1 | 1 |
| 23S rRNA | 1 | 1 | 1 | 1 | 1 | 1 | 3 | 1 | 2 |
| tRNA genes | 38 | 43 | 58 | 39 | 59 | 60 | 53 | 57 | 60 |
| Other RNA genes | 62 | 81 | 88 | 65 | 74 | 90 | 80 | 82 | 97 |
| Protein coding genes with function prediction | 4,812 | 4,621 | 4,516 | 4,753 | 4,608 | 4,598 | 4,804 | 4,611 | 4,606 |
| Protein coding genes without function prediction | 997 | 822 | 797 | 984 | 907 | 886 | 982 | 839 | 875 |
| Protein coding genes with enzymes | 1,300 | 1,355 | 1,341 | 1,331 | 1,339 | 1,339 | 1,312 | 1,337 | 1,339 |
| Protein coding genes without enzymes but with candidate KO based enzymes | 147 | 19 | 26 | 68 | 17 | 11 | 130 | 24 | 13 |
| Protein coding genes connected to Transporter Classification | 832 | 792 | 777 | 810 | 790 | 791 | 809 | 785 | 791 |
| Protein coding genes connected to KEGG pathways | 1,497 | 1,564 | 1,546 | 1,536 | 1,565 | 1,570 | 1,523 | 1,538 | 1,570 |
| not connected to KEGG pathways | 4,312 | 3,879 | 3,767 | 4,201 | 3,950 | 3,914 | 4,263 | 3,912 | 3,911 |
| Protein coding genes connected to KEGG Orthology (KO) | 2,733 | 2,850 | 2,807 | 2,808 | 2,833 | 2,828 | 2,760 | 2,804 | 2,828 |
| not connected to KEGG Orthology (KO) | 3,076 | 2,593 | 2,506 | 2,929 | 2,682 | 2,656 | 3,026 | 2,646 | 2,653 |
| Protein coding genes connected to MetaCyc pathways | 1,272 | 1,325 | 1,311 | 1,300 | 1,313 | 1,313 | 1,287 | 1,312 | 1,313 |
| not connected to MetaCyc pathways | 4,537 | 4,118 | 4,002 | 4,437 | 4,202 | 4,171 | 4,499 | 4,138 | 4,168 |
| Protein coding genes with COGs | 3,839 | 4,012 | 3,927 | 3,923 | 3,965 | 3,979 | 3,927 | 3,949 | 3,978 |
| Protein coding genes with KOGs | 1,930 | 1,902 | 1,880 | 1,918 | 1,910 | 1,908 | 1,983 | 1,929 | 1,915 |
| Protein coding genes with Pfam3 | 4,942 | 4,742 | 4,640 | 4,874 | 4,720 | 4,720 | 4,933 | 4,732 | 4,725 |
| Protein coding genes with TIGRfam3 | 1,727 | 1,753 | 1,740 | 1,757 | 1,771 | 1,775 | 1,766 | 1,744 | 1,774 |
| Protein coding genes with IMG Terms | 365 | 353 | 350 | 357 | 355 | 349 | 353 | 351 | 349 |
| Protein coding genes with IMG Pathways | 181 | 177 | 176 | 178 | 180 | 179 | 181 | 177 | 179 |
| Protein coding genes with IMG Parts List | 142 | 139 | 135 | 141 | 139 | 135 | 136 | 138 | 135 |
| Protein coding genes in paralog clusters | 4674 | 4447 | 4347 | 4655 | 4511 | 4467 | 4698 | 4401 | 4510 |
| Protein coding genes in chromosomal Cassette | 5912 | 5570 | 5463 | 5845 | 5652 | 5638 | 5921 | 5594 | 5643 |
| Chromosomal cassettes | 717 | 532 | 508 | 697 | 556 | 562 | 613 | 560 | 559 |
| Biosynthetic Clusters | 39 | 46 | 38 | 45 | 49 | 50 | 44 | 46 | 43 |
| Genes in Biosynthetic Clusters | 289 | 386 | 332 | 331 | 397 | 410 | 389 | 405 | 369 |
| Fused Protein coding genes | 122 | 119 | 137 | 140 | 139 | 136 | 117 | 136 | 137 |
| Protein coding genes coding signal peptides | 544 | 557 | 554 | 563 | 565 | 578 | 570 | 561 | 577 |
| Protein coding genes coding transmembrane proteins | 1177 | 1235 | 1218 | 1243 | 1210 | 1220 | 1222 | 1207 | 1222 |
| **COG clusters** | 2033 | 2051 | 2046 | 2053 | 2037 | 2039 | 2026 | 2026 | 2039 |
| **KOG clusters** | 841 | 841 | 852 | 852 | 837 | 839 | 840 | 845 | 839 |
| **Pfam clusters** | 2601 | 2566 | 2551 | 2597 | 2547 | 2551 | 2577 | 2547 | 2550 |
| **TIGRfam clusters** | 1345 | 1362 | 1355 | 1369 | 1369 | 1372 | 1365 | 1362 | 1372 |

**Supplementary Table 2.** Detected bacteriocins mined from the nine *P. corrugata* and *P. mediterranea* strains. These genomes along with *P. brassicacearum* subsp. *brassicacearum* NFM421 were blasted with antimicrobial peptides from the BAGEL database (de Jong et al., 2006) and protein hits were recorded.

|  | **Colicin E6**  **(Pyocin S)** | **Carocin D**  **(Pyocin S)** | **Zoocin A**  **(Peptidase M23)** |
| --- | --- | --- | --- |
| ***P. mediterranea*** |  |  |  |
| TEIC1022 | MIALLWPSSLGDSALYSEEQLHNLKQARSRMRLQIEQQADGSLKGYGFYTGQNRDWEMVDVVHLVPRAEHYVADLGDGIELIWTPAVDPSDTLGIPALEAAPQAPPIWIYPPTPMADSIIVDPIYPPEYRDFILVFPADSGVRPVYAVLSVRRKGLAEAGHDYHPAPKTQEIVGFPDLKRGIKKTPVQGGGGLRHRWLDAKGRTIYEWDSSHGELEAYRSSDGSHLGAFDHVTGEQTKAAQKNRNIKKYL |  |  |
| TEIC1105 | VTRQKDIPQVKNPPGGDGHYVTYRDMTPSELAEREAGQLKYEAMLDRQAAYEKERWTKVDKTLLPINGSVFAKSGKLPDGVIDYSNPNGYVPADLVRQYGDLVWLGGRRADASGTVPLKQIGAGAITASLGRLALGGTALEVTGAAVSGSVVTGLLTGMVALLWPSSLGDSALYSEEQLHNLKQARSRMRLQIEQQADGSLKGYGFYTGQNRDWEMVDVVHLVPRAEHYVADLGDGIELIWTPAVDPSDTLGIPALEAAPQAPPIWIYPPTPMADSIIVDPIYPPEYRDFILVFPADSEVRPVYVVVSVRRAGNADHDYYSAPETEEITGFPNLKTAKKKTPKQAGAGLRERWTDLKGRTIYEWDSRHGELEAYRASDGSHLGAFNHETGSGGPGPKKSQHQKVLVRTNGFKTETGLVQQENRNGRRQRVFDRPG |  |  |
| CFBP5404 | VTRQKDIPQVKNPPGGDGHYVTYRDMTPSELAEREAGQLKYEAMLDRQAAYEKERWTKVDKTLLPINGSVFAKSGKLPDGVIDYSNPNGYVPADLVRQYGDLVWLGGRRADASGTVPLKQIGAGAITASLGRLALGGTALEVTGAAVSGSVVTGLLTGMVALLWPSSLGDSALYSEEQLHNLKQARSRMRLQIEQQADGSLKGYGFYTGQNRDWEMVDVVHLVPRAEHYVADLGDGIELIWTPAVDPSDTLGIPALEAAPQAPPIWIYPPTPMADSIIVDPIYPPEYRDFILVFPADSEVRPVYVVVSVRRAGNADHDYYSAPETEEITGFPNLKTAKKKTPKQAGAGLRERWTDLKGRTIYEWDSRHGELEAYRASDGSHLGAFNHETGHQVGPAQKNRNIKKYL |  |  |
| CFBP5444 | VTRQKDIPQVKNPPGGDGHYVTYRDMTPSELAEREAGQLKYEAMLDRQAAYEKERWTKVDKTLLPINGSVFAKSGKLPDGVIDYSNPNGYVPADLVRQYGDLVWLGGRRADASGTVPLKQIGAGAITASLGRLALGGTALEVTGAAVSGSVVTGLLTGMVALLWPSSLGDSALYSEEQLHNLKQARSRMRLQIEQQADGSLKGYGFYTGQNRDWEMVDVVHLVPRAEHYVADLGDGIELIWTPAVDPSDTLGIPALEAAPQAPPIWIYPPTPMADSIIVDPIYPPEYRDFILVFPADSEVRPVYVVVSVRRAGNADHDYYSAPETEEITGFPNLKTAKKKTPKQAGAGLRERWTDLKGRTIYEWDSRHGELEAYRASDGSHLGAFNHETGSGGPGPKKSQHQKVLVRTNGFKTETGLVQQENRNGRRQRVFDRPG |  |  |
| CFBP5447T | 1)VTRQKDIPQVKNPPGGDGHYVTYRDMTPSELAEREAGQLKYEAMLDRQAAYEKERWTKVDKTLLPINGSVFAKSGKLPDGVIDYSNPNGYVPADLVRQYGDLVWLGGRRADASGTVPLKQIGAGAITASLGRLALGGTALEVTGAAVSGSVVTGLLTGMVALLWPSSLGDSALYSEEQLHNLKQARSRMRLQIEQQADGSLKGYGFYTGQNRDWEMVDVVHLVPRAEHYVADLGDGIELIWTPAVDPSDTLGIPALEAAPQAPPIWIYPPTPMADSIIVDPIYPPEYRDFILVFPADSGVRPVYVVVSVRRAGNADHDYYSAPETEEITGFPNLKTAKKKTPKQAGAGLRERWTDLKGRTIYEWDSRHGELEAYRASDGSHLGAFNHETGHQVGPAQKNRNIKKYL  2)MIALLWPSSLGDSSLYSEEQLRNLKQARSRMRLQIEQQTDGSLKGYGFYTGQNRDWEMVDVVHLVPRADHYVADLGDGIELIWTPAVDPSDTLGIPALEAAPQAPPIWIYPPTPMADSIIVDPIYPPEYRDFILVFPADSGVRPVYAVLSVRRKGLAEAGHDYHPAPKTQEIVGFPDLKRGIKKTPVQGGGGLRHRWLDAKGRTIYEWDSSHGELEAYRSSDGSHLGAFDHVTGEQTKAAQKNRNIKKYL |  |  |
| ***P. corrugata*** |  |  |  |
| TEIC1148 |  | MSGTAYSDLGPDTIDYANEWLIESNTDTEKIYRENIKNLKHITDAELSQTRETARKMLPLGVDANNIEARTLTLHLSKARADHQEQVTIANRYYGHDPLTHKGRDPFYKGFDAPDRISQGEYYKAVGKWNISYAAAYEAKFLAEQIKLLEARLVIQNKVVAEASARTAAAAQAKAQADRQRTAAIKARKAAKAEAKRNAKRRRIAAKAAQRQAEIPEARKPVLAATTRPVLASAAIAPALSVIGGSFAADQVIALAITNALRAAAAAVIDTLITLAGPAAAGITALVYTSELGNGDLYVLSVPLSELAPDNTDDLHAIATSRGETHLPVALGSRTIANKTEFIVAATPADSTLAKVPVRLATFDPQENIYKSHSQDAESIGITWTPVVTTDNVSTSLPASIPDVIHYSGAILEIKAGRLDTHPELDRYSFGGFVTVFPVESGIAPLYTVFSNPYEGATTKGEHSGRDFNPDKAGGPIIDLDWRTATVTREGIDTIKLHIARLSPSDANGVMIKRLENILSGDIDVTDTDLRYYTHEIRELERFRNLGLRDDFVPTDDSSYWNNAHTATLEDYKIQDRASLLYTTDALAKADLQDERDYKKMLKEMGQ | MTTEPSKAPPLYPKTHLLAASGIAALLSLALLVFPSSDVEAKKTTLSLELESPAEQLTQEQDAVETAQATNESASSPFAQIDDGSEVTSEAAQGQPPAVEEKPGHHEVIVAKGDTLSTLFEKVGLPAASVHEVLASDKQAREFAQLRHGQKLEFELSPEGQLTSLRTRLSDLETITLTKDDKGYVFNRTTAKPIVRSAYAHGVINSSLSQSAARAGLSHSLTMDMANVFGYDIDFAQDIRQGDEFDVIYEQKMVNGKSVGNGPILSARFTNRGKTYTAVRYINKQGNSSYYTADGNSMRKAFIRTPVDFARISSKFSAGRKHPILNKIRAHKGVDYAAPRGTPIKAAGDGKVLLAGRRGGYGNTVIIQHGNTYRTLYGHMQGFAKGVKTGSTVKQGQVIGYIGTTGLSTGPHLHYEFQVNGVHVDPLGQKLPMADPIAKSERARFMAQSQPLMARMDQEKATLLASSKR |
| CFBP5403 | (partial)LKRGIKKTPVQGGGGLRHRWLDAKGRTIYEWDSSHGELEAYRSSDESHLGAFDHLTGEQTKAAQKNRNIKKYL | MPQEKNILVNRGIPLRRSPSLNLGPGGSIPNGSGFGGMSGTAYSDLGPDTIDYANEWLIESNTDTEKIYRENIKNLKHITDAELSQTRETARKMLPLGVDANNIEARTLTLHLSKARADHQEQVTIANRYYGHDPLTHKGRDPFYKGFDAPDRISQGEYYKAVGKWNISYAAAYEAKFLAEQIKLLEARLVIQNKVVAEASARTAAAAQAKAQADRQRTAAIKARKAAKAEAKRNAKRRRIAAKAAQRQAEIPEARKPVLAATTRPVLASAAIAPALSVIGGSFAADQVIALAITNALRAAAAAVIDTLITLAGPAAAGITALVYTSELGNGDLYVLSVPLSELAPDNTDDLHAIATSRGETHLPVALGSRTIANKTEFIVAATPADSTLAKVPVRLATFDPQENIYKSHSQDAESIGITWTPVVTTDNVSTSLPASIPDVIHYSGAILEIKAGRLDTHPELDRYSFGGFVTVFPVESGIAPLYTVFSNPYEGATTKGEHSGRDFNPDKAGGPIIDLDWRTATVTREGIDTIKLHIARLSPSDANGVMIKRLENILSGDIDVTDTDLRYYTHEIRELERFRNLGLRDDFVPTDDSSYWNNAHTATLEDYKIQDRASLLYTTDALAKADLQDERDYKKMLKEMGQ | MTTEPSKAPPLYPKTHLLAASGIAALLSLALLVFPSSDVEAKKTTLSLELESPAEQLTQEQDAVETAQATNESASSPFAQIDDGSEVTSEAAQGQPPAVEEKPGHHEVIVAKGDTLSTLFEKVGLPAASVHEVLASDKQAREFAQLRHGQKLEFELSPEGQLTSLRTRLSDLETITLTKDDKGYVFNRTTAKPIVRSAYAHGVINSSLSQSAARAGLSHSLTMDMANVFGYDIDFAQDIRQGDEFDVIYEQKMVNGKSVGNGPILSARFTNRGKTYTAVRYINKQGNSSYYTADGNSMRKAFIRTPVDFARISSKFSAGRKHPILNKIRAHKGVDYAAPRGTPIKAAGDGKVLLAGRRGGYGNTVIIQHGNTYRTLYGHMQGFAKGVKTGSTVKQGQVIGYIGTTGLSTGPHLHYEFQVNGVHVDPLGQKLPMADPIAKSERARFMAQSQPLMARMDQEKATLLASSKR |
| CFBP5454 |  | MPQEKNILVNRGIPLRRSPSLNLGPGGSIPNGSGFGGMSGTAYSDLGPDTIDYANEWLIESNTDTEKIYRENIKNLKHITDAELSQTRETARKMLPLGVDANNIEARTLTLHLSKARADHQEQVTIANRYYGHDPLTHKGRDPFYKGFDAPDRISQGEYYKAVGKWNISYAAAYEAKFLAEQIKLLEARLVIQNKVVAEASARTAAAAQAKAQADRQRTAAIKARKAAKAEAKRNAKRRRIAAKAAQRQAEIPEARKPVLAATTRPVLASAAIAPALSVIGGSFAADQVIALAITNALRAAAAAVIDTLITLAGPAAAGITALVYTSELGNGDLYVLSVPLSELAPDNTDDLHAIATSGGETHLPVALGSRTIANKTEFIVAATPADSTLAKVPVRLATFDPQENIYKSHSQDAESIGITWTPVVTTDNVSTSLPASIPDVIHYNGAILEIKAGRLDTHPELDRYSFGGFVTVFPVESGIAPLYTVFSNPYEGATTKGEHSGRDFNPDKAGGPIIDLDWRTATVTREGIDTIKLHIARLSPSDANGVMIKRLENILSGDIDVTDTDLRYYTHEIRELERFRNLGLRDDFVPTDDSSYWNNAHTATLEDYKIQDRASLLYTTDALAKADLQDERDYKKMLKEMGQ | MTTEPSKAPPLYPKTHLLAASGIAALLSLALLVFPSSDVEAKKTTLSLELESPAEQLTQEQDAVETAQATNESAPSPFAQIDDGSEVTSEAAQGQPPAVEEKPGHHEVIVAKGDTLSTLFEKVGLPAASVHEVLASDKQAREFAQLRHGQKLEFELSPEGQLTSLRTRLSDLETITLTKDDKGYVFNRTTAKPIVRSAYAHGVINSSLSQSAARAGLSHSLTMDMANVFGYDIDFAQDIRQGDEFDVIYEQKMVNGKSVGNGPILSARFTNRGKTYTAVRYINKQGNSSYYTADGNSMRKAFIRTPVDFARISSKFSAGRKHPILNKIRAHKGVDYAAPRGTPIKAAGDGKVLLAGRRGGYGNTVIIQHGNTYRTLYGHMQGFAKGVKTGSTVKQGQVIGYIGTTGLSTGPHLHYEFQVNGVHVDPLGQKLPMADPIAKSERARFMAQSQPLMARMDQEKATLLASSKR |
| NCPPB2445T |  | MPQEKNILVNRGIPLRRSPSLNLGPGGSIPNGSGFGGMSGTAYSDLGPDTIDYANEWLIESNTDTEKIYRENIKNLKHITDAELSQTRETARKMLPLGVDANNIEARTLTLHLSKARADHQEQVTIANRYYGHDPLTHKGRDPFYKGFDAPDRISQGEYYKAVGKWNISYAAAYEAKFLAEQIKLLEARLVIQNKVVAEASARTAAAAQAKAQADRQRTAAIKARKAAKAEAKRNAKRRRIAAKAAQRQAEIPEARKPVLAATTRPVLASAAIAPALSVIGGSFAADQVIALAITNALRAAAAAVIDTLITLAGPAAAGITALVYTSELGNGDLYVLSVPLSELAPDNTDDLHAIATSRGETHLPVALGSRTIANKTEFIVAATPADSTLAKVPVRLATFDPQENIYKSHSQDAESIGITWTPVVTTDNVSTSLPASIPDVIHYSGAILEIKAGRLDTHPELDRYSFGGFVTVFPVESGIAPLYTVFSNPYEGATTKGEHSGRDFNPDKAGGPIIDLDWRTATVTREGIDTIKLHIARLSPSDANGVMIKRLENILSGDIDITDTDLRYYTHEIRELERFRNLGLRDDFVPTDDSSYWNNAHTATLEDYKIQDRASLLYTTDALAKADLQDERDYKKMLKEMGQ | MTTEPSKAPPLYPKTHLLAASGIAALLSLALLVFPSSDVEAKKTTLSLELESPAEQLTQEQDAVETAQATNESASSPFAQIDDGSEVTSEAAQGQPPAVEEKPGHHEVIVAKGDTLSTLFEKVGLPAASVHEVLASDKQAREFAQLRHGQKLEFELSPEGQLTSLRTRLSDLETITLTKDDKGYVFNRTIAKPIVRSAYAHGVINSSLSQSAARAGLSHSLTMDMANVFGYDIDFAQDIRQGDEFDVIYEQKMVNGKSVGNGPILSARFTNRGKTYTAVRYINKQGNSSYYTADGNSMRKAFIRTPVDFARISSKFSAGRKHPILNKIRAHKGVDYAAPRGTPIKAAGDGKVLLAGRRGGYGNTVIIQHGNTYRTLYGHMQGFAKGVKTGSTVKQGQVIGYIGTTGLSTGPHLHYEFQVNGVHVDPLGQKLPMADPIAKSERARFMAQSQPLMARMDQEKATLLASSKR |
| F113 |  | VNRGIPRTPSSPDGFGFNARRVSGPDHSDRAYDAVDYALDWLDESNTATEELFNENIRNITHITNTELAKTRAAVAAVVPSGTDALEIELRTLKLQLFKARTDHKEHIAIANLYYGHDPLTHKVQDARYKGFELTGRRGGQRAYYNAIAKWNVSYAAAYEAKFLAEQIKLLDARLTTHNKAIAQANAKAAAEARAKAQAEAKRAAQEQARKAAEAEAKRTAEEQARIARIAATVAAKQAHDAAMALWEAERWADADELEAEEARRQAEVQKARQPARATRTFPVSSSAVVAGPVFTIAGGSLAPNPVTASGITAALRVAVSAIIETVAATALPAVAGFAALVYPSELGNGERYALSVPLSEFVPANTDDLLAVAISKGKINLPVVLGSMTTDDEMAFVVAGADGTSVPSSVPIRLATLDPHSNVYKSSSPDGSSIWMTWTPIIKGTDASTTLPSSVPNILLYEGGSPWATTRWVEKDPELEDYDFGSGFVTVFPVESGIPPIYTVFNSPYEGATTKGEHSGRDFNPEQAGGPILGLDWSTATVTQEGLDAVKLHTGRLNQSDANDVMIERLEKILSGNLDITNTDRRYYTHEIRELERFRALGLADDFSPEPGSPYWNNAHTATLEDYKLQDRELLLYTPDALIKSVLQDERDYQKFLKEISQ |  |

## Supplementary Figures


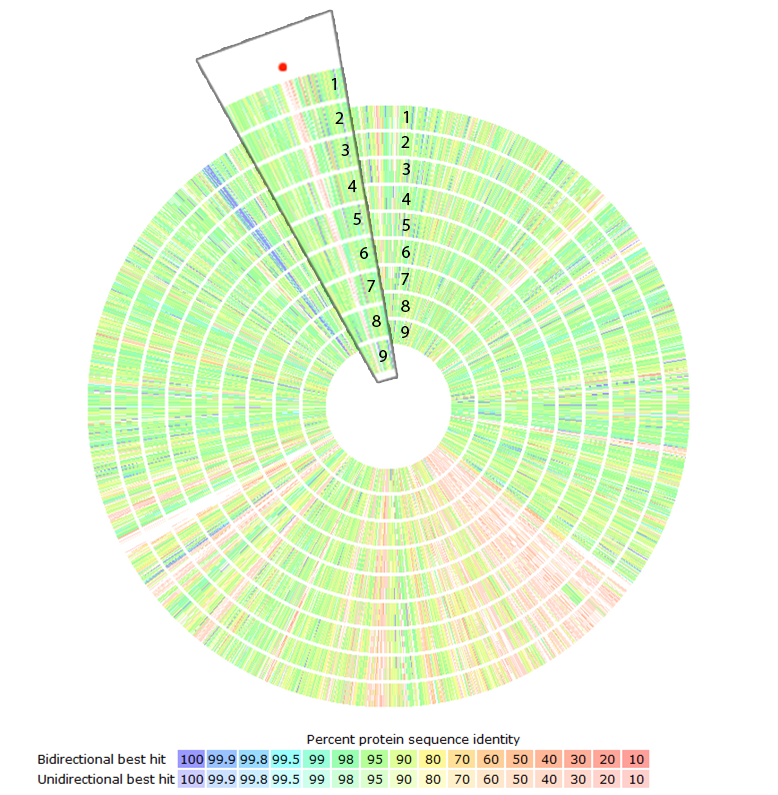


Supplementary Figure 1. Proteome comparison of the nine *Pseudomonas* assembled genomes against the proteome of *P. brassicacearum* subsp. *brassicacearum* NFM421. Each proteome is presented as a closed circle with homology for each protein against the reference genome designated by colored lines. The absence of the Type III secretion system from the *P. corrugata* and *P. mediterranea* genomes is denoted by the red circle. 1: P. *corrugata* CFBP5403, 2: *P. corrugata* CFBP5454, 3: *P. corrugata* NCPPB2445, 4: *P. corrugata* TEIC1148, 5: *P. mediterranea* CFBP5404, 6: P. *mediterranea* CFBP5444, 7: *P. mediterranea* CFBP5447, 8: *P. mediterranea* TEIC1022, 9: *P. mediterranea* TEIC1105.
